# Pointwise Partial Information DecompositionUsing the Specificity and Ambiguity Lattices

**DOI:** 10.3390/e20040297

**Published:** 2018-04-18

**Authors:** Conor Finn, Joseph T. Lizier

**Affiliations:** 1 Complex Systems Research Group and Centre for Complex Systems, Faculty of Engineering & IT, The University of Sydney, NSW 2006, Australia; 2 CSIRO Data61, Marsfield NSW 2122, Australia

**Keywords:** mutual information, pointwise information, information decomposition, unique information, redundant information, complementary information, redundancy, synergy, 89.70.Cf, 89.75.Fb, 05.65.+b, 87.19.lo

## Abstract

What are the distinct ways in which a set of predictor variables can provide information about a target variable? When does a variable provide unique information, when do variables share redundant information, and when do variables combine synergistically to provide complementary information? The redundancy lattice from the partial information decomposition of Williams and Beer provided a promising glimpse at the answer to these questions. However, this structure was constructed using a much criticised measure of redundant information, and despite sustained research, no completely satisfactory replacement measure has been proposed. In this paper, we take a different approach, applying the axiomatic derivation of the redundancy lattice to a single realisation from a set of discrete variables. To overcome the difficulty associated with signed pointwise mutual information, we apply this decomposition separately to the unsigned entropic components of pointwise mutual information which we refer to as the specificity and ambiguity. This yields a separate redundancy lattice for each component. Then based upon an operational interpretation of redundancy, we define measures of redundant specificity and ambiguity enabling us to evaluate the partial information atoms in each lattice. These atoms can be recombined to yield the sought-after multivariate information decomposition. We apply this framework to canonical examples from the literature and discuss the results and the various properties of the decomposition. In particular, the pointwise decomposition using specificity and ambiguity satisfies a chain rule over target variables, which provides new insights into the so-called two-bit-copy example.

## 1. Introduction

The aim of information decomposition is to divide the total amount of information provided by a set of predictor variables, about a target variable, into atoms of partial information contributed either individually or jointly by the various subsets of the predictors. Suppose that we are trying to predict a target variable *T*, with discrete state space T, from a pair of predictor variables $S_{1}$ and $S_{2}$, with discrete state spaces $S_{1}$ and $S_{2}$. The mutual information $I(S_{1};T)$ quantifies the information $S_{1}$ individually provides about *T*. Similarly, the mutual information $I(S_{2};T)$ quantifies the information $S_{2}$ individually provides about *T*. Now consider the joint variable $S_{1,2}$ with the state space $S_{1}\times S_{2}$. The (joint) mutual information $I(S_{1,2};T)$ quantifies the total information $S_{1}$ and $S_{2}$ together provide about *T*. Although Shannon’s information theory provides the prior three measures of information, there are four possible ways $S_{1}$ and $S_{2}$ could contribute information about *T*: the predictor $S_{1}$ could uniquely provide information about *T*; or the predictor $S_{2}$ could uniquely provide information about *T*; both $S_{1}$ and $S_{2}$ could both individually, yet redundantly, provide the same information about *T*; or the predictors $S_{1}$ and $S_{2}$ could synergistically provide information about *T* which is not available in either predictor individually. Thus we have the following underdetermined set of equations,

(1) $$\begin{array}{ccc} I(S_{1,2};T) & =R(S_{1},S_{2}\to T) & +U(S_{1}\backslash S_{2}\to T)+U(S_{2}\backslash S_{1}\to T)+C(S_{1},S_{2}\to T),\\ I(S_{1};T) & =R(S_{1},S_{2}\to T) & +U(S_{1}\backslash S_{2}\to T),\\ I(S_{2};T) & =R(S_{1},S_{2}\to T) & +U(S_{2}\backslash S_{1}\to T), \end{array}$$

where $U(S_{1}\backslash S_{2}\to T)$ and $U(S_{2}\backslash S_{1}\to T)$ are the unique information provided by $S_{1}$ and $S_{2}$ respectively, $R(S_{1},S_{2}\to T)$ is the redundant information, and $C(S_{1},S_{2}\to T)$ is the synergistic or complementary information. (The directed notation is utilise here to emphasis the privileged role of the variable *T*.) Together, the equations in (1) form the bivariate information decomposition. The problem is to define one of the unique, redundant or complementary information—something not provided by Shannon’s information theory—in order to uniquely evaluate the decomposition.

Now suppose that we are trying to predict a target variable *T* from a set of *n* finite state predictor variables $S=\{S_{1},\ldots ,S_{n}\}$. In this general case, the aim of information decomposition is to divide the total amount of information $I(S_{1},\ldots ,S_{n};T)$ into atoms of partial information contributed either individually or jointly by the various subsets of S. But what are the distinct ways in which these subsets of predictors might contribute information about the target? Multivariate information decomposition is more involved than the bivariate information decomposition because it is not immediately obvious how many atoms of information one needs to consider, nor is it clear how these atoms should relate to each other. Thus the general problem of information decomposition is to provide both a structure for multivariate information which is consistent with the bivariate decomposition, and a way to uniquely evaluate the atoms in this general structure.

In the remainder of Section 1, we will introduce an intriguing framework called partial information decomposition (PID), which aims to address the general problem of information decomposition, and highlight some of the criticisms and weaknesses of this framework. In Section 2, we will consider the underappreciated pointwise nature of information and discuss the relevance of this to the problem of information decomposition. We will then propose a modified pointwise partial information decomposition (PPID), but then quickly repudiate this approach due to complications associated with decomposing the signed pointwise mutual information. In Section 3, we will discuss circumventing this issue by examining information on a more fundamental level, in terms of the unsigned entropic components of pointwise mutual information which we refer to as the specificity and the ambiguity. Then in Section 4—the main section of this paper—we will introduce the PPID using the specificity and ambiguity lattices and the measures of redundancy in Definitions 1 and 2. In Section 5, we will apply this framework to a number of canonical examples from the PID literature, discuss some of the key properties of the decomposition, and compare these to existing approaches to information decomposition. Section 6 will conclude the main body of the paper. Appendix A contains discussions regarding the so-called two-bit-copy problem in terms of Kelly gambling, Appendix B contains many of the technical details and proofs, while Appendix B contains some more examples.

### 1.1. Notation

The following notational conventions are observed throughout this article:

*T*, T, *t*, $t^{c}$ denote the *target* variable, event space, event and complementary event respectively;
*S*, S, *s*, $s^{c}$ denote the *predictor* variable, event space, event and complementary event respectively;
S, s represent the *set* of *n* predictor variables $\left\{S_{1},\ldots ,S_{n}\right\}$ and events $\left\{s_{1},\ldots ,s_{n}\right\}$ respectively;
$T^{t}$, $S^{s}$ denote the *two-event partition* of the event space, i.e., $T^{t}=\left\{t,t^{c}\right\}$ and $S^{s}=\left\{s,s^{c}\right\}$;
H(T), I(S;T) uppercase function names be used for *average* information-theoretic measures;
h(t), i(s,t) lowercase function names be used for *pointwise* information-theoretic measures.

When required, the following index conventions are observed:

$s^{1}$, $s^{2}$, $t^{1}$, $t^{2}$ superscripts distinguish between different *different events* in a variable;
$S_{1}$, $S_{2}$, $T_{1}$, $T_{2}$ subscripts distinguish between *different variables*;
$S_{1,2}$, $s_{1,2}$ multiple superscripts represent *joint variables* and *joint events*.

Finally, to be discussed in more detail when appropriate, consider the following:

$A_{1},\ldots ,A_{k}$ *sources* are sets of predictor variables, i.e., $A_{i}\in P_{1}\left(S\right)$ where $P_{1}$ is the power set without ∅;
$a_{1},\ldots ,a_{k}$ *source events* are sets of predictor events, i.e., $a_{i}\in P_{1}\left(s\right)$.

### 1.2. Partial Information Decomposition

The *partial information decomposition* (PID) of Williams and Beer [1,2] was introduced to address the problem of multivariate information decomposition. The approach taken is appealing as rather than speculating about the structure of multivariate information, Williams and Beer took a more principled, axiomatic approach. They start by considering potentially overlapping subsets of S called sources, denoted $A_{1},\ldots ,A_{k}$. To examine the various ways these sources might contain the same information, they introduce three axioms which “any reasonable measure for redundant information [$I_{\cap }$] should fulfil” ([3], p. 3502). Note that the axioms appear explicitly in [2] but are discussed in [1] as mere properties; a published version of the axioms can be found in [4].

**W&B** **Axiom** **1** (Commutativity)**.** *Redundant information is invariant under any permutation σ of sources,*

$$I_{\cap }\left(A_{1},\ldots ,A_{k}\to T\right)=I_{\cap }\left(\sigma \left(A_{1}\right),\cdots ,\sigma \left(A_{k}\right)\to T\right).$$

**W&B** **Axiom** **2** (Monotonicity)**.** *Redundant information decreases monotonically as more sources are included,*

$$I_{\cap }\left(A_{1},\ldots ,A_{k-1}\to T\right)\leq I_{\cap }\left(A_{1},\ldots ,A_{k}\to T\right)$$

*with equality if $A_{k}⊇A_{i}$ for any $A_{i}\in \left\{A_{1},\ldots ,A_{k-1}\right\}$.*

**W&B** **Axiom** **3** (Self-redundancy)**.** *Redundant information for a single source $A_{i}$ equals the mutual information,*

$$I_{\cap }\left(A_{i}\to T\right)=I\left(A_{i}\;;T\right).$$

These axioms are based upon the intuition that redundancy should be analogous to the set- theoretic notion of intersection (which is commutative, monotonically decreasing and idempotent). Crucially, Axiom 3 ties this notion of redundancy to Shannon’s information theory. In addition to these three axioms, there is an (implicit) axiom assumed here known as *local positivity* [5], which is the requirement that all atoms be non-negative. Williams and Beer [1,2] then show how these axioms reduce the number of sources to the collection of sources such that no source is a superset of any other. These remaining sources are called *partial information atoms* (PI atoms). Each PI atom corresponds to a distinct way the set of predictors S can contribute information about the target *T*. Furthermore, Williams and Beer show that these PI atoms are partially ordered and hence form a lattice which they call the *redundancy lattice*. For the bivariate case, the redundancy lattice recovers the decomposition (1), while in the multivariate case it provides a meaningful structure for decomposition of the total information provided by an arbitrary number of predictor variables.

While the redundancy lattice of PID provides a structure for multivariate information decomposition, it does not uniquely determine the value of the PI atoms in the lattice. To do so requires a definition of a measure of redundant information which satisfies the above axioms. Hence, in order to complete the PID framework, Williams and Beer simultaneously introduced a measure of redundant information called $I_{\min}$ which quantifies redundancy as the minimum information that any source provides about a target event *t*, averaged over all possible events from *T*. However, not long after its introduction $I_{\min}$ was heavily criticised. Firstly, $I_{\min}$ does not distinguish between “whether different random variables carry the *same* information or just the *same amount* of information” ([5], p. 269; see also [6,7]). Secondly, $I_{\min}$ does not possess the target chain rule introduced by Bertschinger et al. [5] (under the name left chain rule). This latter point is problematic as the target chain rule is a natural generalisation of the chain rule of mutual information—i.e., one of the fundamental, and indeed characterising, properties of information in Shannon’s theory [8,9].

These issues with $I_{\min}$ prompted much research attempting to find a suitable replacement measure compatible with the PID framework. Using the methods of information geometry, Harder et al. [6] focused on a definition of redundant information called $I_{\mathrm{red}}$ (see also [10]). Bertschinger et al. [11] defined a measure of unique information $\tilde{UI}$ based upon the notion that if one variable contains unique information then there must be some way to exploit that information in a decision problem. Griffith and Koch [12] used an entirely different motivation to define a measure of synergistic information $S_{\mathrm{VK}}$ whose decomposition transpired to be equivalent to that of $\tilde{UI}$ [11]. Despite this effort, none of these proposed measures are entirely satisfactory. Firstly, just as for $I_{\min}$, none of these proposed measures possess the target chain rule. Secondly, these measures are not compatible with the PID framework in general, but rather are only compatible with PID for the special case of bivariate predictors, i.e., the decomposition (1). This is because they all simultaneously satisfy the Williams and Beers axioms, local positivity, and the *identity property* introduced by Harder et al. [6]. In particular, Rauh et al. [13] proved that no measure satisfying the identity property and the Williams and Beer Axioms 1–3 can yield a non-negative information decomposition beyond the bivariate case of two predictor variables. In addition to these proposed replacements for $I_{\min}$, there is also a substantial body of literature discussing either PID, similar attempts to decompose multivariate information, or the problem of information decomposition in general [3,4,5,7,10,13,14,15,16,17,18,19,20,21,22,23,24,25,26,27,28]. Furthermore, the current proposals have been applied to various problems in neuroscience [29,30,31,32,33,34]. Nevertheless (to date), there is no generally accepted measure of redundant information that is entirely compatible with PID framework, nor has any other well-accepted multivariate information decomposition emerged.

To summarise the problem, we are seeking a meaningful decomposition of the information provided an arbitrarily large set of predictor variables about a target variable, into atoms of partial information contributed either individually or jointly by the various subsets of the predictors. Crucially, the redundant information must capture when two predictor variables are carrying the same information about the target, not merely the same amount of information. Finally, any proposed measure of redundant information should satisfy the target chain rule so that net redundant information can be consistently computed for consistently for multiple target events.

## 2. Pointwise Information Theory

Both the entropy and mutual information can be derived from first principles as fundamentally *pointwise* quantities which measure the information content of individual events rather than entire variables. The pointwise entropy h(t)=−logp(t) quantifies the information content of a single event *t*, while the pointwise mutual information

(2) $$i\left(s;t\right)=\log\frac{p(t|s)}{p(t)}=\log\frac{p(s,t)}{p(s)p(t)}=\log\frac{p(s|t)}{p(s)},$$

quantifies the information provided by *s* about *t*, or vice versa. To our knowledge, these quantities were first considered by Woodward and Davies [35,36] who noted that the average form of Shannon’s entropy “tempts one to enquire into other simpler methods of derivation [of the per state entropy]” ([35], p. 51). Indeed, they went on to show that the pointwise entropy and pointwise mutual information can be derived from two axioms concerning the addition of the information provided by the occurrence of individual events [36]. Fano [9] further formalised this pointwise approach by deriving both quantites from four postulates which “should be satisfied by a useful measure of information” ([9], p. 31). Taking the expectation of these pointwise quantities over all events recovers the average entropy $H\left(T\right)=\langle h(t)\rangle$ and average mutual information $I\left(S;T\right)=\langle i(s;t)\rangle$ first derived by Shannon [8]. Although both approaches arrive at the same average quantities, Shannon’s treatment obfuscates the pointwise nature of the fundamental quantities. In contrast, the approach of Woodward, Davis and Fano makes this pointwise nature manifestly obvious.

It is important to note that, in contrast to the average mutual information, the pointwise mutual information is not non-negative. Positive pointwise information corresponds to the predictor event *s* raising the probability p(t|s) relative to the prior probability p(t). Hence when the event *t* occurs it can be said that the event *s* was *informative* about the event *t*. Conversely, negative pointwise information corresponds to the event *s* lowering the posterior probability p(t|s) relative to the prior probability p(t). Hence when the event *t* occurs we can say that the event *s* was *misinformative* about the event *t*. (Not to be confused with disinformation, i.e., intentionally misleading information.) Although a source event *s* may be misinformative about a particular target event *t*, a source event *s* is never misinformative about the target variable *T* since the pointwise mutual information averaged over all target realisations is non-negative [9]. The information provided by *s* is helpful for predicting *T* on average; however, in certain instances this (typically helpful) information is misleading in that it lowers p(t|s) relative to p(t)—typically helpful information which subsequently turns out to be misleading is misinformation.

Finally, before continuing, there are two points to be made about the terminology used to describe pointwise information. Firstly, in certain literature (typically in the context of time-series analysis), the word *local* is used instead of pointwise, e.g., [4,18]. Secondly, in contemporary information theory, the word average is generally omitted while the pointwise quantities are explicitly prefixed; however, this was not always the accepted convention. Woodward [35] and Fano [9] both referred to pointwise mutual information as the *mutual information* and then explicitly prefixed the *average mutual information*. To avoid confusion, we will always prefix both pointwise and average quantities.

### 2.1. Pointwise Information Decomposition

Now that we are familiar with pointwise nature of information, suppose that we have a discrete realisation from the joint event space $T\times S_{1}\times S_{2}$ consisting of the target event *t* and predictor events $s_{1}$ and $s_{2}$. The pointwise mutual information $i(s_{1};t)$ quantifies the information provided individually by $s_{1}$ about *t*, while the pointwise mutual information $i(s_{2};t)$ quantifies the information provided individually by $s_{2}$ about *t*. The pointwise joint mutual information $i(s_{1,2};t)$ quantifies the total information provided jointly by $s_{1}$ and $s_{2}$ about *t*. In correspondence with the (average) bivariate decomposition (1), consider the pointwise bivariate decomposition, first suggested by Lizier et al. [4],

(3) $$\begin{array}{ccc} i(s_{1,2};t) & =r(s_{1},s_{2}\to t) & +u(s_{1}\backslash s_{2}\to t)+u(s_{2}\backslash s_{1}\to t)+c(s_{1},s_{2}\to t),\\ i(s_{1};t) & =r(s_{1},s_{2}\to t) & +u(s_{1}\backslash s_{2}\to t),\\ i(s_{2};t) & =r(s_{1},s_{2}\to t) & +u(s_{2}\backslash s_{1}\to t). \end{array}$$

Note that the lower case quantities denote the pointwise equivalent of the corresponding upper case quantities in (1). This decomposition could be considered for every discrete realisation on the support of the joint distribution $P(S_{1},S_{2},T)$. Hence, consider taking the expectation of these pointwise atoms over all discrete realisations,

(4) $$\begin{array}{cccc} U(S_{1}\backslash S_{2}\to T) & =\langle u(s_{1}\backslash s_{2}\to t)\rangle , & R(S_{1},S_{2}\to T) & =\langle r(s_{1},s_{2}\to t)\rangle ,\\ U(S_{2}\backslash S_{1}\to T) & =\langle u(s_{2}\backslash s_{1}\to t)\rangle , & C(S_{1},S_{2}\to T) & =\langle c(s_{1},s_{2}\to t)\rangle . \end{array}$$

Since the expectation is a linear operation, this will recover the (average) bivariate decomposition (1). Equation (3) for every discrete realisation, together with (1) and (4) form the bivariate pointwise information decomposition. Just as in (1), these equations are underdetermined requiring a separate definition of either the pointwise unique, redundant or complementary information for uniqueness. (Defining an average atom is sufficient for a unique bivariate decomposition (1), but still leaves the pointwise decomposition (3) within each realisation underdetermined).

### 2.2. Pointwise Unique

Now consider applying this pointwise information decomposition to the probability distribution *Pointwise Unique* (PwUnq) in Table 1. In PwUnq, observing 0 in either of $S_{1}$ or $S_{2}$ provides zero information about the target *T*, while complete information about the outcome of *T* is obtained by observing 1 or a 2 in either predictor. The probability distribution is structured such that in each of the four realisations, one predictor provides complete information while the other predictor provides zero information—the two predictors never provide the same information about the target which is justified by noting that one of the two predictors always provides zero pointwise information.

Given that redundancy is supposed to capture the same information, it seems reasonable to assume there must be zero pointwise redundant information for each realisation. This assumption is made without any measure of pointwise redundant information; however, no other possibility seems justifiable. This assertion is used to determine the pointwise redundant information terms in Table 1. Then using the pointwise information decomposition (3), we can then evaluate the other pointwise atoms of information in Table 1. Finally using (4), we get that there is zero (average) redundant information, and 12 bit of (average) unique information from each predictor. From the pointwise perspective, the only reasonable conclusion seems to be that the predictors in PwUnq must contain only unique information about the target.

However, in contrast to the above, $I_{\min}$, $I_{\mathrm{red}}$, $\tilde{UI}$, and $S_{\mathrm{VK}}$ all say that the predictors in PwUnq contain no unique information, rather only 12 bit of redundant information plus 12 bit of complementary information. This problem, which will be referred to as the *pointwise unique problem*, is a consequence of the fact that these measures all satisfy Assumption (∗) of Bertschinger et al. [11], which (in effect) states that the unique and redundant information should only depend on the marginal distributions $P(S_{1},T)$ and $P(S_{2},T)$. In particular, any measure which satisfies Assumption (∗) will yield zero unique information when $P(S_{1},T)$ is isomorphic to $P(S_{2},T)$, as is the case for PwUnq. (Here, isomorphic should be taken to mean isomorphic probability spaces, e.g., [37], p. 27 or [38], p. 4.) It arises because Assumption (∗) (and indeed the operational interpretation the led to its introduction) does not respect the pointwise nature of information. This operational view does not take into account the fact that individual events $s_{1}$ and $s_{2}$ may provide different information about the event *t*, even if the probability distributions $P(S_{1},T)$ and $P(S_{2},T)$ are the same. Hence, we contend that for any measure to capture the same information (not merely the same amount), it must respect the pointwise nature of information.

### 2.3. Pointwise Partial Information Decomposition

With the pointwise unique problem in mind, consider constructing an information decomposition with the pointwise nature of information as an inherent property. Let $a_{1},\ldots ,a_{k}$ be potentially intersecting subsets of the predictor events $s=\{s_{1},\ldots ,s_{n}\}$, called source events. Now consider rewriting the Williams and Beer axioms in terms of a measure of pointwise redundant information $i_{\cap }$ where the aim is to deriving a *pointwise partial information decomposition* (PPID).

**PPID** **Axiom** **1** (Symmetry)**.** *Pointwise redundant information is invariant under any permutation σ of source events,*

$$i_{\cap }\left(a_{1},\ldots ,a_{k}\to t\right)=i_{\cap }\left(\sigma \left(a_{1}\right),\cdots ,\sigma \left(a_{k}\right)\to T\right).$$

**PPID** **Axiom** **2** (Monotonicity)**.** *Pointwise redundant information decreases monotonically as more source events are included,*

$$i_{\cap }\left(a_{1},\ldots ,a_{k-1}\to t\right)\leq i_{\cap }\left(a_{1},\ldots ,a_{k}\to t\right)$$

*with equality if $a_{k}⊇a_{i}$ for any $a_{i}\in \left\{a_{1},\ldots ,a_{k-1}\right\}$.*

**PPID** **Axiom** **3** (Self-redundancy)**.** *Pointwise redundant information for a single source event $a_{i}$ equals the pointwise mutual information,*

$$i_{\cap }\left(a_{i}\to t\right)=i\left(a_{i}\;;t\right).$$

It seems that the next step should be to define some measure of pointwise redundant information which is compatible with these PPID axioms; however, there is a problem—the pointwise mutual information is not non-negative. While this would not be an issue for the examples like PwUnq, where none of the source events provide negative pointwise information, it is an issue in general (e.g., see RdnErr in Section 5.4). The problem is that set-theoretic intuition behind Axiom 2 (monotonicity) makes little sense when considering signed measures like the pointwise mutual information.

Given the desire to address the pointwise unique problem, there is a need to overcome this issue. Ince [18] suggested that the set-theoretic intuition is only valid when all source events provide either positive or negative pointwise information. Ince contends that information and misinformation are “fundamentally different” ([18], p. 11) and that the set-theoretic intuition should be admitted in the difficult to interpret situations where both are present. We however, will take a different approach—one which aims to deal with these difficult to interpret situations whilst preserving the set-theoretic intuition that redundancy corresponds to overlapping information.

By way of a preview, we first consider precisely how an event $s_{1}$ provides information about an event *t* by the means of two distinct types of probability mass exclusion. We show how considering the process in this way naturally splits the pointwise mutual information into particular entropic components, and how one can consider redundancy on each of these components separately. Splitting the signed pointwise mutual information into these unsigned entropic components circumvents the above issue with Axiom 2 (monotonicity). Crucially, however, by deriving these entropic components from the probability mass exclusions, we retain the set-theoretic intuition of redundancy—redundant information will correspond to overlapping probability mass exclusions in the two-event partition $T^{t}=\left\{t,t^{c}\right\}$.

## 3. Probability Mass Exclusions and the Directed Components of Pointwise Mutual Information

**Figure 1:**
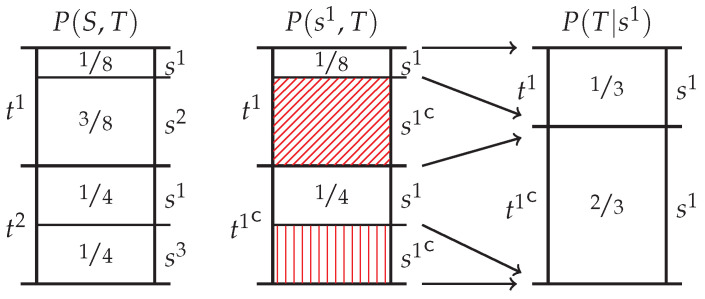
Sample probability mass diagrams, which use length to represent the probability mass of each joint event from T×S. (**Left**) the joint distribution P(S,T); (**Middle**) The occurrence of the event $s^{1}$ leads to exclusions of the complementary event ${s^{1}}^{c}$ which consists of two elementary event, i.e., ${s^{1}}^{c}=\left\{s^{2},s^{3}\right\}$. This leaves the probability mass $P(s^{1},T)$ remaining. The exclusion of the probability mass $p({s^{1}}^{c},t^{1})$ was misinformative since the event $t^{1}$ did occur. By convention, misinformative exclusions will be indicated with diagonal hatching. On the other hand, the exclusion of the probability mass $p({t^{1}}^{c},{s^{1}}^{c})$ was informative since the complementary event ${t^{1}}^{c}$ did not occur. By convention, informative exclusions will be indicated with horizontal or vertical hatching; (**Right**) this remaining probability mass can be normalised yielding the conditional distribution $P(T|s^{1})$.

By definition, the pointwise information provided by *s* about *t* is associated with a change from the prior p(t) to the posterior p(t|s). As we explored from first principles in Finn and Lizier [39], this change is a consequence of the *exclusion* of probability mass in the target distribution P(T) induced by the occurrence of the event *s* and inferred via the joint distribution P(S,T). To be specific, when the event *s* occurs, one knows that the complementary event $s^{c}=\left\{S\backslash s\right\}$ did not occur. Hence one can *exclude* the probability mass in the joint distribution P(S,T) associated with the complementary event, i.e., exclude $P(s^{c},T)$, leaving just the probability mass P(s,T) remaining. The new target distribution P(T|s) is evaluated by normalising this remaining probability mass. In [39] we introduced *probability mass diagrams* in order to visually explore the exclusion process. Figure 1 provides an example of such a diagram. Clearly, this process is merely a description of the definition of conditional probability. Nevertheless, we content that by viewing the change from the prior to the posterior in this way—by focusing explicitly on the exclusions rather than the resultant conditional probability—the vague intuition that redundancy corresponds to overlapping information becomes more apparent. This point will elaborated upon in Section 3.3. However, in order to do so, we need to first discuss the two distinct types of probability mass exclusion (which we do in Section 3.1) and then relate these to information-theoretic quantities (which we do in Section 3.2).

### 3.1. Two Distinct Types of Probability Mass Exclusions

In [39] we examined the two distinct types of probability mass exclusions. The difference between the two depends on where the exclusion occurs in the target distribution P(T) and the particular target event *t* which occurred. *Informative exclusions* are those which are confined to the probability mass associated with the set of elementary events in the target distribution which *did not occur*, i.e., exclusions confined to the probability mass of the complementary event $p(t^{c})$. They are called such because the pointwise mutual information i(s;t) is a monotonically increasing function of the total size of these exclusions $p(t^{c})$. By convention, informative exclusions are represented on the probability mass diagrams by horizontal or vertical lines. On the other hand, the *misinformative exclusion* is confined to the probability mass associated with the elementary event in the target distribution which *did occur*, i.e., an exclusion confined to p(t). It is referred to as such because the pointwise mutual information i(s;t) is a monotonically decreasing function of the size of this type of exclusion p(t). By convention, misinformative exclusions are represented on the probability mass diagrams by diagonal lines.

Although an event *s* may exclusively induce either type of exclusion, in general both types of exclusion are present simultaneously. The distinction between the two types of exclusions leads naturally to the following question—can one decompose the pointwise mutual information i(s;t) into a positive informational component associated with the informative exclusions, and a negative informational component associated with the misinformative exclusions? This question is considered in detail in Section 3.2. However, before moving on, there is a crucial observation to be made about the pointwise mutual information which will have important implications for the measure of redundant information to be introduced later.

**Remark** **1.** The pointwise mutual information i(s;t) depends only on the size of informative and misinformative exclusions. In particular, it does not depend on the apportionment of the informative exclusions across the set of elementary events contained in the complementary event $t^{c}$.

In other words, whether the event *s* turns out to be net informative or misinformative about the event *t*—whether i(s;t) is positive or negative—depends on the size of the two types of exclusions; but, to be explicit, does *not* depend on the distribution of the informative exclusion across the set of target events which did not occur. This remark will be crucially important when it comes to providing the operational interpretation of redundant information in Section 3.3. (It is also further discussed in terms of Kelly gambling [40] in Appendix A).

### 3.2. The Directed Components of Pointwise Information: Specificity and Ambiguity

We return now to the idea that one might be able to decompose the pointwise mutual information into a positive and negative component associated with the informative amd misinformative exclusions respectively. In [39] we proposed four postulates for such a decomposition. Before stating the postulates, it is important to note that although there is a “surprising symmetry” ([41], p. 23) between the information provided by *s* about *t* and the information provided by *t* about *s*, there is nothing to suggest that the components of the decomposition should be symmetric—indeed the intuition behind the decomposition only makes sense when considering the information is considered in a directed sense. As such, directed notation will be used to explicitly denote the information provided by *s* about *t*.

**Postulate** **1** (Decomposition)**.** The pointwise information provided by s about t can be decomposed into two non-negative components, such that $i\left(s;t\right)=i_{+}\left(s\to t\right)-i_{-}\left(s\to t\right)$.

**Postulate** **2** (Monotonicity)**.** For all fixed p(s,t) and $p(s^{c},t)$, the function $i_{+}\left(s\to t\right)$ is a monotonically increasing, continuous function of $p(t^{c},s^{c})$. For all fixed $p(t^{c},s)$ and $p(t^{c},s^{c})$, the function $i_{-}\left(s\to t\right)$ is a monotonically increasing continuous function of $p(s^{c},t)$. For all fixed p(s,t) and $p(t^{c},s)$, the functions $i_{+}\left(s\to t\right)$ and $i_{-}\left(s\to t\right)$ are monotonically increasing and decreasing functions of $p(t^{c},s^{c})$, respectively.

**Postulate** **3** (Self-Information)**.** An event cannot misinform about itself, $i_{+}\left(s\to s\right)=i\left(s;s\right)=-\log p\left(s\right)$.

**Postulate** **4** (Chain Rule)**.** *The functions $i_{+}\left(s_{1,2}\to t\right)$ and $i_{-}\left(s_{1,2}\to t\right)$ satisfy a chain rule, i.e.,*

$$\begin{array}{cc} i_{+}\left(s_{1,2}\to t\right) & =i_{+}\left(s_{1}\to t\right)+i_{+}\left(s_{2}\to t|s_{1}\right)\\ & =i_{+}\left(s_{2}\to t\right)+i_{+}\left(s_{1}\to t|s_{2}\right),\\ i_{-}\left(s_{1,2}\to t\right) & =i_{-}\left(s_{1}\to t\right)+i_{-}\left(s_{2}\to t|s_{1}\right)\\ & =i_{-}\left(s_{2}\to t\right)+i_{-}\left(s_{1}\to t|s_{2}\right) \end{array}$$

In Finn and Lizier [39], we proved that these postulates lead to the following forms which are unique up to the choice of the base of the logarithm in the mutual information in Postulates 1 and 3,

(5) $$\begin{array}{ccc} i^{+}\left(s_{1}\to t\right) & =h(s_{1}) & =-\log p(s_{1}), \end{array}$$

(6) $$\begin{array}{ccc} i^{+}\left(s_{1}\to t|s_{2}\right) & =h(s_{1}|s_{2}) & =-\log p(s_{1}|s_{2}), \end{array}$$

(7) $$\begin{array}{ccc} i^{+}\left(s_{1,2}\to t\right) & =h(s_{1,2}) & =-\log p(s_{1,2}), \end{array}$$

(8) $$\begin{array}{ccc} i^{-}\left(s_{1}\to t\right) & =h(s_{1}|t) & =-\log p(s_{1}|t), \end{array}$$

(9) $$\begin{array}{ccc} i^{-}\left(s_{1}\to t|s_{2}\right)\; & =h(s_{1}|t,s_{2})\; & =-\log p(s_{1}|t,s_{2}), \end{array}$$

(10) $$\begin{array}{ccc} i^{-}\left(s_{1,2}\to t\right) & =h(s_{1,2}|t) & =-\log p(s_{1,2}|t). \end{array}$$

That is, the Postulates 1–4 uniquely decompose the pointwise information provided by *s* about *t* into the following entropic components,

(11) $$\begin{array}{cc} i(s;t) & =i^{+}\left(s\to t\right)-i^{-}\left(s\to t\right)\\ & =h(s)-h(s|t). \end{array}$$

Although the decomposition of mutual information into entropic components is well-known, it is non-trivial that Postulates 1 and 3, based on the size of the two distinct types of probability mass exclusions, lead to this particular form, but not i(s;t)=h(t)−h(t|s) or i(s;t)=h(s)+h(t)−h(s,t).

It is important to note that although the original motivation was to decompose the pointwise mutual information into separate components associated with informative and misinformative exclusion, the decomposition (11) does not quite possess this direct correspondence:

- The positive informational component $i^{+}\left(s\to t\right)$ does not depend on *t* but rather only on *s*. This can be interpreted as follows: the less likely *s* is to occur, the more specific it is when it does occur, the greater the total amount of probability mass excluded $p(s^{c})$, and the greater the potential for *s* to inform about *t* (or indeed any other target realisation).
- The negative informational component $i^{-}\left(s\to t\right)$ depends on both *s* and *t*, and can be interpreted as follows: the less likely *s* is to coincide with the event *t*, the more uncertainty in *s* given *t*, the greater size of the misinformative probability mass exclusion $p(s^{c},t)$, and therefore the greater the potential for *s* to misinform about *t*.

In other words, although the negative informational component $i^{-}\left(s\to t\right)$ does correspond directly to the size of the misinformative exclusion $p(s^{c},t)$, the positive informational component $i^{+}\left(s\to t\right)$ does not correspond directly to the size of the informative exclusion $p(t^{c},s^{c})$. Rather, the positive informational component $i^{+}\left(s\to t\right)$ corresponds to the *total* size of the probability mass exclusions $p(s^{c})$, which is the sum of the sum of the informative and misinformative exclusions. For the sake of brevity, the positive informational component $i^{+}\left(s\to t\right)$ will be referred to as the *specificity*, while the negative informational component $i^{-}\left(s\to t\right)$ will be referred to as the *ambiguity*. The term ambiguity is due to Shannon: “[equivocation] measures the average ambiguity of the received signal” ([42], p. 67). Specificity is an antonym of ambiguity and the usage here is inline with the definition since the more specific an event *s*, the more information it could provide about *t* after the ambiguity is taken into account.

### 3.3. Operational Interpretation of Redundant Information

Arguing about whether one piece of information differs from another piece of information is nonsensical without some kind of unambiguous definition of what it means for two pieces of information to be the same. As such, Bertschinger et al. [11] advocate the need to provide an operational interpretation of what it means for information to be unique or redundant. This section provides our operational definition of what it means for information to be the same. This definition provides a concrete interpretation of what it means for information to be redundant in terms of overlapping probability mass exclusions.

The operational interpretation of redundancy adopted here is based upon the following idea: since the pointwise information is ultimately derived from probability mass exclusions, the *same information* must induce the *same exclusions*. More formally, the information provided by a set of predictor events $s_{1},\ldots ,s_{k}$ about a target event *t* must be the same information if each source event induces the same exclusions with respect to the two-event partition $T^{t}=\left\{t,t^{c}\right\}$. While this statement makes the motivational intuition clear, it is not yet sufficient to serve as an operational interpretation of redundancy: there is no reference to the two distinct types of probability mass exclusions, the specific reference to the pointwise event space $T^{t}$ has not been explained, and there is no reference to the fact the exclusions from each source may differ in size.

Informative exclusions are fundamentally different from misinformative exclusions and hence each type of exclusion should be compared separately: informative exclusions can overlap with informative exclusions, and misinformative exclusions can overlap with misinformative exclusions. In information-theoretic terms, this means comparing the specificity and the ambiguity of the sources separately—i.e., considering a measure of redundant specificity and a separate measure of redundant ambiguity. Crucially, these quantities (being pointwise entropies) are unsigned meaning that the difficulties associated with Axiom 2 (Monotonicity) and signed pointwise mutual information in Section 2.3 will not be an issue here.

The specific reference to the two-event partition $T^{t}$ in the above statement is based upon Remark 1 and is crucially important. The pointwise mutual information does not depend on the apportionment of the informative exclusions across the set of events which did not occur, hence the pointwise redundant information should not depend on this apportionment either. In other words, it is immaterial if two predictor events $s_{1}$ and $s_{2}$ exclude different elementary events within the target complementary event $t^{c}$ (assuming the probability mass excluded is equal) since with respect to the realised target event *t* the difference between the exclusions is only semantic. This has important implications for the comparison of exclusions from different predictor events. As the pointwise mutual information depends on, and only depends on, the size of the exclusions, then the only sensible comparison is a comparison of size. Hence, the common or overlapping exclusion must be the smallest exclusion. Thus, consider the following operational interpretation of redundancy:

**Operational** **Interpretation** (Redundant Specificity)**.** The redundant specificity between a set of predictor events $s_{1},\ldots ,s_{n}$ is the specificity associated with the source event which induces the smallest total exclusions.

**Operational** **Interpretation** (Redundant Ambiguity)**.** The redundant ambiguity between a set of predictor events $s_{1},\ldots ,s_{n}$ is the ambiguity associated with the source event which induces the smallest misinformative exclusion.

### 3.4. Motivational Example

**Figure 2:**
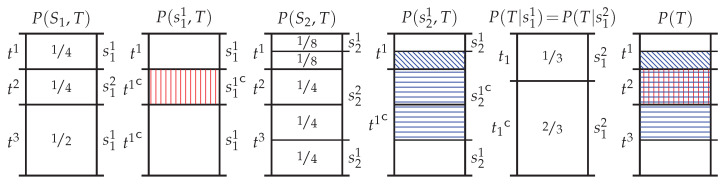
Sample probability mass diagrams for two predictors $S_{1}$ and $S_{2}$ to a given target *T*. Here events in the two different predictor spaces provide the same amount of pointwise information about the target event, log243 bits, since $P\left(T|s_{1}^{1}\right)=P\left(T|s_{2}^{1}\right)$, although each excludes different sections of the target distribution P(T). Since they both provide the same amount of information, is there a way to characterise what information the additional unique exclusions from the event $s_{2}^{1}$ are providing?

To motivate the above operational interpretation, and in particular the need to treat the specificity separately to the ambiguity, consider Figure 2. In this pointwise example, two different predictor events provide the same amount of pointwise information since $P\left(T|s_{1}^{1}\right)=P\left(T|s_{2}^{1}\right)$, and yet the information provided by each event is in some way different since each excludes different sections of the target distribution P(T). In particular, $s_{1}^{1}$ and $s_{2}^{1}$ both preclude the target event $t^{2}$, while $s_{2}^{1}$ additionally excludes probability mass associated with target events $t^{1}$ and $t^{3}$. From the perspective of the pointwise mutual information the events $s_{1}^{1}$ and $s_{2}^{1}$ seem to be providing the same information as

(12) i(s11→t1)=i(s21→t1)=log43bit.

However, from the perspective of the specificity and the ambiguity it can be seen that information is being provided in different ways since

(13) i+(s11→t1)=log43bit,i−(s11→t1)=0bit,i+(s21→t1)=log83bit,i−(s21→t1)=1bit.

Now consider the problem of decomposing information into its unique, redundant and complementary components. Figure 2 shows where exclusions induced by $s_{1}^{1}$ and $s_{2}^{1}$ overlap where they both exclude the target event $t^{2}$ which is an informative exclusion. This is the only exclusion induced by $s_{1}^{1}$ and hence all of the information associated with this exclusion must be redundantly provided by the event $s_{2}^{1}$. Without any formal framework, consider taking the redundant specificity and redundant ambiguity,

(14) r+(s11,s21→t1)=i+(s11→t1)=log43bit,

(15) $$\begin{array}{cccc} r^{-}\left(s_{1}^{1},s_{2}^{1}\to t_{1}\right) & =i^{-}\left(s_{1}^{1}\to t^{1}\right)= & 0 & \;\mathrm{bit}. \end{array}$$

This would mean that the event $s_{2}^{1}$ provides the following unique specificity and unique ambiguity,

(16) $$\begin{array}{ccc} u^{+}\left(s_{1}^{1}\backslash s_{2}^{1}\to t^{1}\right) & =i^{+}\left(s_{1}^{1}\to t^{1}\right)-r^{+}\left(s_{1}^{1},s_{2}^{1}\to t^{1}\right) & =1\;\mathrm{bit}, \end{array}$$

(17) $$\begin{array}{ccc} u^{-}\left(s_{1}^{1}\backslash s_{2}^{1}\to t^{1}\right) & =i^{-}\left(s_{1}^{1}\to t^{1}\right)-r^{-}\left(s_{1}^{1},s_{2}^{1}\to t^{1}\right) & =1\;\mathrm{bit}. \end{array}$$

The redundant specificity log 43 bit accounts for the overlapping informative exclusion of the event $t^{2}$. The unique specificity and unique ambiguity from $s_{2}^{1}$ are associated with its non-overlapping informative and misinformative exclusions; however, both of these 1 bit and hence, on net, $s_{2}^{1}$ is no more informative than $s_{1}^{1}$. Although obtained without a formal framework, this example highlights a need to consider the specificity and ambiguity rather than merely the pointwise mutual information.

## 4. Pointwise Partial Information Decomposition Using Specificity and Ambiguity

Based upon the argumentation of Section 3, consider the following axioms:

**Axiom** **1** (Symmetry)**.** *Pointwise redundant specificity $i_{\cap }^{+}$ and pointwise redundant ambiguity $i_{\cap }^{-}$ are invariant under any permutation σ of source events,*

$$\begin{array}{cc} i_{\cap }^{+}\left(a_{1},\cdots ,a_{k}\to t\right) & =i_{\cap }^{+}\left(\sigma \left(a_{1}\right),\cdots ,\sigma \left(a_{k}\right)\to t\right),\\ i_{\cap }^{-}\left(a_{1},\cdots ,a_{k}\to t\right) & =i_{\cap }^{-}\left(\sigma \left(a_{1}\right),\cdots ,\sigma \left(a_{k}\right)\to t\right). \end{array}$$

**Axiom** **2** (Monotonicity)**.** *Pointwise redundant specificity $i_{\cap }^{+}$ and pointwise redundant ambiguity $i_{\cap }^{-}$ decreases monotonically as more source events are included,*

$$\begin{array}{cc} i_{\cap }^{+}\left(a_{1},\cdots ,a_{k-1},a_{k}\to t\right) & \leq i_{\cap }^{+}\left(a_{1},\cdots ,a_{k-1}\to t\right),\\ i_{\cap }^{-}\left(a_{1},\cdots ,a_{k-1},a_{k}\to t\right) & \leq i_{\cap }^{-}\left(a_{1},\cdots ,a_{k-1}\to t\right). \end{array}$$

*with equality if $a_{k}⊇a_{i}$ for any $a_{i}\in \left\{a_{1},\ldots ,a_{k-1}\right\}$.*

**Axiom** **3** (Self-redundancy)**.** *Pointwise redundant specificity $i_{\cap }^{+}$ and pointwise redundant ambiguity $i_{\cap }^{-}$ for a single source event $a_{i}$ equals the specificity and ambiguity respectively,*

$$\begin{array}{ccc} i_{\cap }^{+}\left(a_{i}\to t\right) & =i^{+}\left(a_{i}\to t\right) & =h(a_{i}),\\ i_{\cap }^{-}\left(a_{i}\to t\right) & =i^{-}\left(a_{i}\to t\right) & =h(a_{i}|t). \end{array}$$

**Figure 3:**
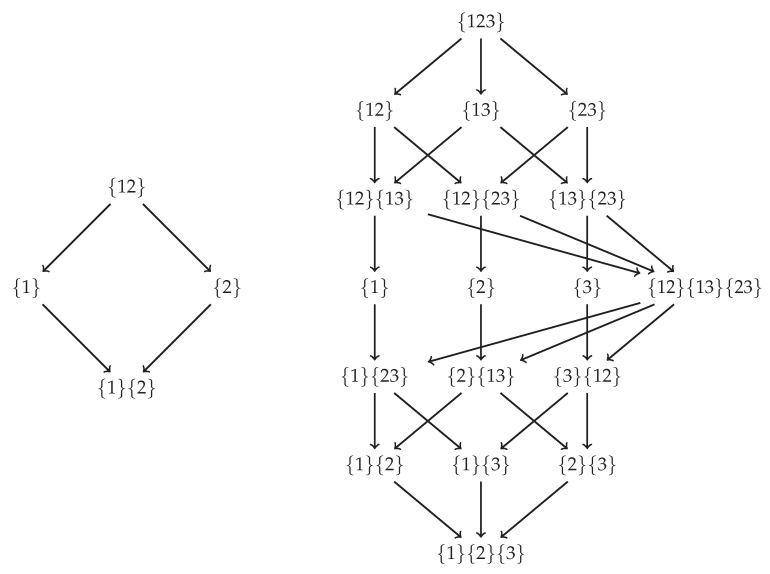
The lattice induced by the partial order ⪯ (A15) over the sources A(s) (A14). (**Left**) the lattice for $s=\{s_{1},s_{2}\}$; (**Right**) the lattice for $s=\{s_{1},s_{2},s_{3}\}$. See Appendix B for further details. Each node corresponds to the self-redundancy (Axiom 3) of a source event, e.g., {1} corresponds to the source event $\left\{\{s_{1}\}\right\}$, while {12,13} corresponds to the source event $\left\{\left\{s_{1,2}\right\},\left\{s_{1,3}\right\}\right\}$. Note that the specificity and ambiguity lattices share the same structure as the redundancy lattice of partial information decomposition (PID) (cf. Figure 2 in [1]).

As shown in Appendix B.1, Axioms 1–3 induce two lattices—namely the *specificity lattice* and *ambiguity lattice*—which are depicted in Figure 3. Furthermore, each lattice is defined for every discrete realisation from $P(S_{1},\ldots ,S_{n},T)$. The redundancy measures $i_{\cap }^{+}$ or $i_{\cap }^{-}$ can be thought of as a cumulative information functions which integrate the specificity or ambiguity uniquely contributed by each node as one moves up each lattice. Finally, just as in PID, performing a Möbius inversion over each lattice yielding the unique contributions of specificity and ambiguity from each sources event.

Similarly to PID, the specificity and ambiguity lattices provide a structure for information decomposition, but unique evaluation requires a separate definition of redundancy. However, unlike PID (or even PPID), this evaluation requires both a definition of pointwise redundant specificity and pointwise redundant ambiguity. Before providing these definitions, it is helpful to first see how the specificity and ambiguity lattices can be used to decompose multivariate information in the now familiar bivariate case.

### 4.1. Bivariate PPID Using the Specificity and Ambiguity

Consider again the bivariate case where the aim is to decompose the information provided by $s_{1}$ and $s_{2}$ about *t*. The specificity lattice can be used to decompose the pointwise specificity,

(18) $$\begin{array}{cc} i^{+}\left(s_{1,2}\to t\right) & =r^{+}\left(s_{1},s_{2}\to t\right)+u^{+}\left(s_{1}\backslash s_{2}\to t\right)+u^{+}\left(s_{2}\backslash s_{1}\to t\right)+c^{+}\left(s_{1},s_{2}\to t\right),\\ i^{+}\left(s_{1}\to t\right) & =r^{+}\left(s_{1},s_{2}\to t\right)+u^{+}\left(s_{1}\backslash s_{2}\to t\right),\\ i^{+}\left(s_{2}\to t\right) & =r^{+}\left(s_{1},s_{2}\to t\right)+u^{+}\left(s_{2}\backslash s_{1}\to t\right); \end{array}$$

while the ambiguity lattice can be used to decompose the pointwise ambiguity,

(19) $$\begin{array}{cc} i^{-}\left(s_{1,2}\to t\right) & =r^{-}\left(s_{1},s_{2}\to t\right)+u^{-}\left(s_{1}\backslash s_{2}\to t\right)+u^{-}\left(s_{2}\backslash s_{1}\to t\right)+c^{-}\left(s_{1},s_{2}\to t\right),\\ i^{-}\left(s_{1}\to t\right) & =r^{-}\left(s_{1},s_{2}\to t\right)+u^{-}\left(s_{1}\backslash s_{2}\to t\right),\\ i^{-}\left(s_{2}\to t\right) & =r^{-}\left(s_{1},s_{2}\to t\right)+u^{-}\left(s_{2}\backslash s_{1}\to t\right). \end{array}$$

These equations share the same structural form as (3) only now decompose the specificity and the ambiguity rather than the pointwise mutual information, e.g., $r^{+}\left(s_{1},s_{2}\to t\right)$ denotes the redundant specificity while $u^{-}\left(s_{1}\backslash s_{2}\to t\right)$ denoted the unique ambiguity from $s_{1}$. Just as in for (3), this decomposition could be considered for every discrete realisation on the support of the joint distribution $P(S_{1},S_{2},T)$.

There are two ways one can be combine these values. Firstly, in a similar manner to (4), one could take the expectation of the atoms of specificity, or the atoms of ambiguity, over all discrete realisations yielding the average PI atoms of specificity and ambiguity,

(20) $$\begin{array}{cccc} U^{+}\left(S_{1}\backslash S_{2}\to T\right) & =\langle u^{+}\left(s_{1}\backslash s_{2}\to t\right)\rangle , & U^{-}\left(S_{1}\backslash S_{2}\to T\right) & =\langle u^{-}\left(s_{1}\backslash s_{2}\to t\right)\rangle ,\\ U^{+}\left(S_{2}\backslash S_{1}\to T\right) & =\langle u^{+}\left(s_{2}\backslash s_{1}\to t\right)\rangle , & U^{-}\left(S_{2}\backslash S_{1}\to T\right) & =\langle u^{-}\left(s_{2}\backslash s_{1}\to t\right)\rangle ,\\ R^{+}\left(S_{1},S_{2}\to T\right) & =\langle r^{+}\left(s_{1},s_{2}\to t\right)\rangle , & R^{-}\left(S_{1},S_{2}\to T\right) & =\langle r^{-}\left(s_{1},s_{2}\to t\right)\rangle ,\\ C^{+}\left(S_{1},S_{2}\to T\right) & =\langle c^{+}\left(s_{1},s_{2}\to t\right)\rangle . & C^{-}\left(S_{1},S_{2}\to T\right) & =\langle c^{-}\left(s_{1},s_{2}\to t\right)\rangle . \end{array}$$

Alternatively, one could subtract the pointwise unique, redundant and complementary ambiguity from the pointwise unique, redundant and complementary specificity yielding the pointwise unique, pointwise redundant and pointwise complementary information, i.e., recover the atoms from PPID,

(21) $$\begin{array}{cc} r(s_{1},s_{2}\to t) & =r^{+}\left(s_{1},s_{2}\to t\right)-r^{-}\left(s_{1},s_{2}\to t\right),\\ u(s_{1}\backslash s_{2}\to t) & =u^{+}\left(s_{1}\backslash s_{2}\to t\right)-u^{-}\left(s_{1}\backslash s_{2}\to t\right),\\ u(s_{2}\backslash s_{1}\to t) & =u^{+}\left(s_{2}\backslash s_{1}\to t\right)-u^{-}\left(s_{2}\backslash s_{1}\to t\right),\\ c(s_{1},s_{2}\to t) & =c^{+}\left(s_{1},s_{2}\to t\right)-c^{-}\left(s_{1},s_{2}\to t\right). \end{array}$$

Both (20) and (21) are linear operations, hence one could perform both of these operations (in either order) to obtain the average unique, average redundant and average complementary information, i.e., recover the atoms from PID,

(22) $$\begin{array}{cc} R(S_{1},S_{2}\to T) & =R^{+}\left(S_{1},S_{2}\to T\right)-R^{-}\left(S_{1},S_{2}\to T\right),\\ U(S_{1}\backslash S_{2}\to T) & =U^{+}\left(S_{1}\backslash S_{2}\to T\right)-U^{-}\left(S_{1}\backslash S_{2}\to T\right),\\ U(S_{2}\backslash S_{1}\to T) & =U^{+}\left(S_{2}\backslash S_{1}\to T\right)-U^{-}\left(S_{2}\backslash S_{1}\to T\right),\\ C(S_{1},S_{2}\to T) & =C^{+}\left(S_{1},S_{2}\to T\right)-C^{-}\left(S_{1},S_{2}\to T\right). \end{array}$$

### 4.2. Redundancy Measures on the Specificity and Ambiguity Lattices

Now that we have a structure for our information decomposition, there is a need to provide a definition of the pointwise redundant specificity and pointwise redundant ambiguity. However, before attempting to provide such a definition, there is a need to consider Remark 1 and the operational interpretation of in Section 3.3. In particular, the pointwise redundant specificity $i_{\cap }^{+}$ and pointwise redundant ambiguity $i_{\cap }^{-}$ should only depend on the size of informative and misinformative exclusions. They should not depend on the apportionment of the informative exclusions across the set of elementary events contained in the complementary event $t^{c}$. Formally, this requirement will be enshrined via the following axiom.

**Axiom** **4** (Two-event Partition)**.** The pointwise redundant specificity $i_{\cap }^{+}$ and pointwise redundant ambiguity $i_{\cap }^{-}$ are functions of the probability measures on the two-event partitions $A_{1}^{a_{1}}\times T^{t},\cdots ,A_{k}^{a_{k}}\times T^{t}$.

Since the pointwise redundant specificity $i_{\cap }^{+}$ is specificity associated with the source event which induces the smallest total exclusions, and pointwise redundant ambiguity $i_{\cap }^{-}$ is the ambiguity associated with the source event which induces the smallest misinformative exclusion, consider the following definitions.

**Definition** **1.** *The pointwise redundant specificity is given by*

(23) $$r_{min}^{+}\left(a_{1},\cdots ,a_{k}\to t\right)=\min_{a_{i}}\;i^{+}\left(a_{i}\to t\right)=\min_{a_{i}}\;h\left(a_{i}\right).$$

**Definition** **2.** *The pointwise redundant ambiguity is given by*

(24) $$r_{min}^{-}\left(a_{1},\cdots ,a_{k}\to t\right)=\min_{a_{i}}\;i^{-}\left(a_{i}\to t\right)=\min_{a_{j}}\;h\left(a_{j}|t\right).$$

**Theorem** **1.** The definitions of $r_{min}^{+}$ and $r_{min}^{-}$ satisfy Axioms 1–4.

**Theorem** **2.** The redundancy measures $r_{min}^{+}$ and $r_{min}^{-}$ increase monotonically on the $\langle A(s),⪯\rangle$.

**Theorem** **3.** The atoms of partial specificity $\pi^{+}$ and partial ambiguity $\pi^{-}$ evaluated using the measures $r_{min}^{+}$ and $r_{min}^{-}$ on the specificity and ambiguity lattices (respectively), are non-negative.

Appendix B.2 contains the proof of Theorems 1–3 and further relevant consideration of Defintions 1 and 2. As in (20), one can take the expectation of the either the pointwise redundant specificity $r_{\min}^{+}$ or the pointwise redundant ambiguity $r_{\min}^{-}$ to get the average redundant specificity $R_{\min}^{+}$ or the average redundant ambiguity $R_{\min}^{-}$. Alternatively, just as in (21), one can recombine the pointwise redundant specificity $r_{\min}^{+}$ and the pointwise redundant ambiguity $r_{\min}^{-}$ to get the pointwise redundant information $r_{\min}$. Finally, as per (22), one could perform both of these (linear) operations in either order to obtain the average redundant information $R_{\min}$. Note that while Theorem 3 proves that the atoms of partial specificity $\pi^{+}$ and partial ambiguity $\pi^{-}$ are non-negative, it is trivial to see that $r_{\min}$ could be negative since when source events can redundantly provide misinformation about a target event. As shown in the following theorem, $R_{\min}$ can also be negative.

**Theorem** **4.** The atoms of partial average information Π evaluated by recombining and averaging $\pi^{\pm }$ are not non-negative.

This means that the measure $R_{\min}$ does not satisfy local positivity. Nonetheless the negativity of $R_{\min}$ is readily explainable in terms of the operational interpretation of Section 3.3, as will be discussed further in Section 5.4. However, failing to satisfy local positivity does mean that $r_{\min}$ and $R_{\min}$ do not satisfy the *target monotonicity* property first discussed in Bertschinger et al. [5]. Despite this, as the following theorem shows, the measures do satisfy the target chain rule.

**Theorem** **5** (Pointwise Target Chain Rule)**.** *Given the joint target realisation $t_{1,2}$, the pointwise redundant information $r_{min}$ satisfies the following chain rule,*

(25) $$\begin{array}{cc} r_{min}\left(a_{1},\ldots ,a_{k}\to t_{1,2}\right) & =r_{min}\left(a_{1},\ldots ,a_{k}\to t_{1}\right)+r_{min}\left(a_{1},\ldots ,a_{k}\to t_{2}|t_{1}\right),\\ & =r_{min}\left(a_{1},\ldots ,a_{k}\to t_{2}\right)+r_{min}\left(a_{1},\ldots ,a_{k}\to t_{1}|t_{2}\right). \end{array}$$

The proof of the last theorem is deferred to Appendix B.3. Note that since the expectation is a linear operation, Theorem 5 also holds for the average redundant information $R_{\min}$. Furthermore, as these results apply to any of the source events, the target chain rule will hold for any of the PPI atoms, e.g., (21), and any of the PI atoms, e.g., (22). However, no such rule holds for the pointwise redundant specificity or ambiguity. The specificity depends only on the predictor event, i.e., does not depend on the target events. As such, when an increasing number of target events are considered, the specificity remains unchanged. Hence, a target chain rule cannot hold for the specificity, or the ambiguity alone.

## 5. Discussion

PPID using the specificity and ambiguity takes the ideas underpinning PID and applies them on a pointwise scale while circumventing the monotonicity issue associated with the signed pointwise mutual information. This section will explore the various properties of the decomposition in an example driven manner and compare the results to the most widely-used measures from the existing PID literature. (Further examples can be found in Appendix C.) The following shorthand notation will be utilised in the figures throughout this section:

$$\begin{array}{cccccc} i_{1}^{+} & =i^{+}\left(s_{1}\to t\right), & i_{2}^{+} & =i^{+}\left(s_{2}\to t\right), & i_{1,2}^{+} & =i^{+}\left(s_{1,2}\to t\right),\\ i_{1}^{-} & =i^{-}\left(s_{1}\to t\right), & i_{2}^{-} & =i^{-}\left(s_{2}\to t\right), & i_{1,2}^{-} & =i^{-}\left(s_{1,2}\to t\right), \end{array}$$

$$\begin{array}{cccccccc} u_{1}^{+} & =u^{+}\left(s_{1}\backslash s_{2}\to t\right), & u_{2}^{+} & =u^{+}\left(s_{2}\backslash s_{1}\to t\right), & r^{+} & =r^{+}\left(s_{1},s_{2}\to t\right), & c^{+} & =c^{+}\left(s_{1},s_{2}\to t\right),\\ u_{1}^{-} & =u^{-}\left(s_{1}\backslash s_{2}\to t\right), & u_{2}^{-} & =u^{-}\left(s_{2}\backslash s_{1}\to t\right), & r^{-} & =r^{-}\left(s_{1},s_{2}\to t\right), & c^{-} & =c^{-}\left(s_{1},s_{2}\to t\right). \end{array}$$

### 5.1. Comparison to Existing Measures

A similar approach to the decomposition presented in this paper is due to Ince [18], who also sought to define a pointwise information decomposition. Despite the similarity in this regard, the redundancy measure $I_{\mathrm{ccs}}$ presented in [18] approaches the pointwise monotonicity problem of Section 2.3 in a different way to the decomposition presented in this paper. Specifically, $I_{\mathrm{ccs}}$ aims to utilise the pointwise co-information as a measure of pointwise redundant information since it “quantifies the set-theoretic overlap of the two univariate [pointwise] information values” ([18], p. 14). There are, however, difficulties with this approach. Firstly (unlike the average mutual information and the Shannon inequalities), there are no inequalities which support this interpretation of pointwise co-information as the set-theoretic overlap of the univariate pointwise information terms—indeed, both the univariate pointwise information and the pointwise co-information are signed measures. Secondly, the pointwise co-information conflates the pointwise redundant information with the pointwise complementary information, since by (3) we have that

(26) $$co-i\left(s_{1};s_{2};t\right)=i\left(s_{1};t\right)+i\left(s_{2};t\right)-i\left(s_{1,2},t\right)=r(s_{1},s_{2}\to t)-c(s_{1},s_{2}\to t).$$

Aware of these difficulties, Ince defines $I_{\mathrm{ccs}}$ such that it only interprets the pointwise co-information as a measure of set-theoretic overlap in the case where all three pointwise information terms have the same sign, arguing that these are the only situations which admit a clear interpretation in terms of a common change in surprisal. In the other difficult to interpret situations, $I_{\mathrm{ccs}}$ defines the pointwise redundant information to be zero. This approach effectively assumes that $c(s_{1},s_{2}\to t)=0$ in (26) when $i(s_{1};t)$, $i(s_{2};t)$ and $co-i(s_{1};s_{2};t)$ all have the same sign.

In a subsequent paper, Ince [19] also presented a partial entropy decomposition which aims to decompose multivariate entropy rather than multivariate information. As such, this decomposition is more similar to PPID using specificity and ambiguity than Ince’s aforementioned decomposition. Although similar in this regard, the measure of pointwise redundant entropy $H_{\mathrm{cs}}$ presented in [19] takes a different approach to the one presented in this paper. Specifically, $H_{\mathrm{cs}}$ also uses the pointwise co-information as a measure of set-theoretic overlap and hence as a measure of pointwise redundant entropy. As the pointwise entropy is unsigned, the difficulties are reduced but remain present due to the signed pointwise co-information. In a manner similar to $I_{\mathrm{ccs}}$, Ince defines $H_{\mathrm{cs}}$ such that it only interprets the pointwise co-information as a measure of set-theoretic overlap when it is positive. As per $I_{\mathrm{ccs}}$, this effectively assumes that $c(s_{1},s_{2}\to t)=0$ in (26) when all information terms have the same sign. When the pointwise co-information is negative, $H_{\mathrm{cs}}$ simply ignores the co-information by defining the pointwise redundant information to be zero. In contrast to both of Ince’s approaches, PPID using specificity and ambiguity does not dispose of the set-theoretic intuition in these difficult to interpret situations. Rather, our approach considers the notion of redundancy in terms of overlapping exclusions—i.e., in terms of the underlying, unsigned measures which are amenable to a set-theoretic interpretation.

The measures of pointwise redundant specificity $r_{\min}^{+}$ and pointwise redundant ambiguity $r_{\min}^{-}$, from Definitions 1 and 2 are also similar to both the minimum mutual information $I_{\mathrm{mmi}}$ [17] and the original PID redundancy measure $I_{\min}$ [1]. Specifically, all three of these approaches consider the redundant information to be the minimum information provided about a target event *t*. The difference is that $I_{\min}$ applies this idea to the sources $A_{1},\ldots ,A_{k}$, i.e., to collections of entire predictor variables from S, while $r_{\min}^{\pm }$ apply this notion to the source events $a_{1},\ldots ,a_{k}$, i.e., to collections of predictor events from s. In other words, while the measure $I_{\min}$ can be regarded as being semi-pointwise (since it considers the information provided by the variables $S_{1},\ldots ,S_{n}$ about an event *t*), the measures $r_{\min}^{\pm }$ are fully pointwise (since they consider the information provided by the events $s_{1},\ldots ,s_{n}$ about an event *t*). This difference in approach is most apparent in the probability distribution PwUnq—unlike PID, PPID using the specificity and ambiguity respects the pointwise nature of information, as we will see in Section 5.3.

PPID using specificity and ambiguity also share certain similarities with the bivariate PID induced by the measure $\tilde{UI}$ of Bertschinger et al. [11]. Firstly, Axiom 4 can be considered to be a pointwise adaptation of their Assumption (∗), i.e., the measures $r_{\min}^{\pm }$ depend only on the marginal distributions $P(S_{1},T)$ and $P(S_{2},T)$ with respect to the two-event partitions $S_{1}^{s_{1}}\times T^{t}$ and $S_{2}^{s_{2}}\times T^{t}$. Secondly, in PPID using specificity and ambiguity, the only way one can only decide if there is complementary information $c(s_{1},s_{2}\to t)$ is by knowing the joint distribution $P(S_{1},S_{2},T)$ with respect to the joint two-event partitions $S_{1}^{s_{1}}\times S_{2}^{s_{2}}\times T^{t}$. This is (in effect) a pointwise form of their Assumption (∗∗). Thirdly, by definition $r_{\min}^{\pm }$ are given by the minimum value that any one source event provides. This is the largest possible value that one could take for these quantities whilst still requiring that the unique specificity and ambiguity be non-negative. Hence, within each discrete realisation, $r_{\min}^{\pm }$ minimise the unique specificity and ambiguity whilst maximising the redundant specificity and ambiguity. This is similar to $\tilde{UI}$ which minimises the (average) unique information while still satisfying Assumption (∗). Finally, note that since the measure $S_{\mathrm{VK}}$ produces a bivariate decomposition which is equivalent to that of $\tilde{UI}$ [11], the same similarities apply between PPID using specificity and ambiguity and the decomposition induced by $S_{\mathrm{VK}}$ from Griffith and Koch [12].

### 5.2. Probability Distribution X*or*

**Figure 4:**
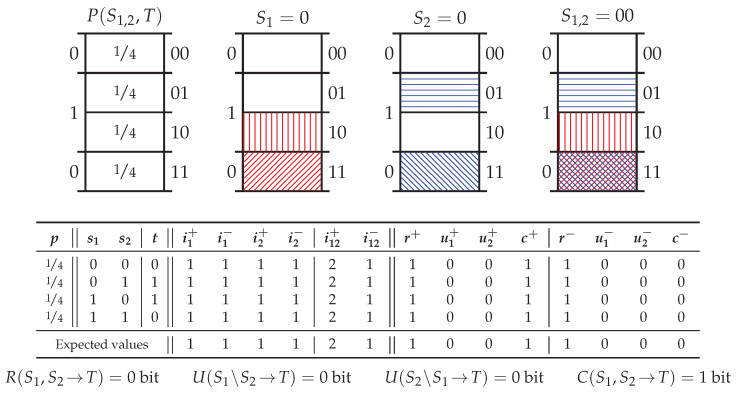
Example Xor. (**Top**) probability mass diagrams for the realisation $\left(S_{1}=0,S_{2}=0,T=0\right)$; (**Middle**) For each realisation, the pointwise specificity and pointwise ambiguity has been evaluated using (5) and (8) respectively. The pointwise redundant specificity and pointwise redundant ambiguity are then determined using (23) and (24). The decomposition is calculated using (18) and (19). The expected specificity and ambiguity are calculated with (20); (**Bottom**) The average information is given by (22). As expected, Xor yields 1 bit of complementary information.

Figure 4 shows the canonical example of synergy, *exclusive-or* (Xor) which considers two independently distributed binary predictor variables $S_{1}$ and $S_{2}$ and a target variable $T=S_{1}\;\mathrm{XOR}\;S_{2}$. There are several important points to note about the decomposition of Xor. Firstly, despite providing zero pointwise information, an individual predictor event does indeed induce exclusions. However, the informative and misinformative exclusions are perfectly balanced such that the posterior (conditional) distribution is equal to the prior distribution, e.g., see the red coloured exclusions induced by $S_{1}=0$ in Figure 4. In information-theoretic terms, for each realisation, the pointwise specificity equals 1 bit since half of the total probability mass remains while the pointwise ambiguity also equals 1 bit since half of the probability mass associated with the event which subsequently occurs (i.e., T=0), remains. These are perfectly balanced such that when recombined, as per (11), the pointwise mutual information is equal to 0 bit, as one would expect.

Secondly, $S_{1}=0$ and $S_{2}=0$ both induce the same exclusions with respect to the target pointwise event space $T^{T=0}$. Hence, as per the operational interpretation of redundancy adopted in Section 3.3, there is 1 bit of pointwise redundant specificity and 1 bit of pointwise redundant ambiguity in each realisation. The presence of (a form of) redundancy in Xor is novel amongst the existing measures in the PID literature. (Ince [19] also identifies a form of redundancy in Xor.) Thirdly, despite the presence of this redundancy, recombining the atoms of pointwise specificity and ambiguity for each realisation, as per (21), leaves only one non-zero PPI atom: namely the pointwise complementary information $c(s_{1},s_{2}\to t)$ = 1 bit. Furthermore, this is true for every pointwise realisation and hence, by (22), the only non-zero PI atom is the average complementary information $C(S_{1},S_{2}\to T)$ = 1 bit.

### 5.3. Probability Distribution *PwUnq*

**Figure 5:**
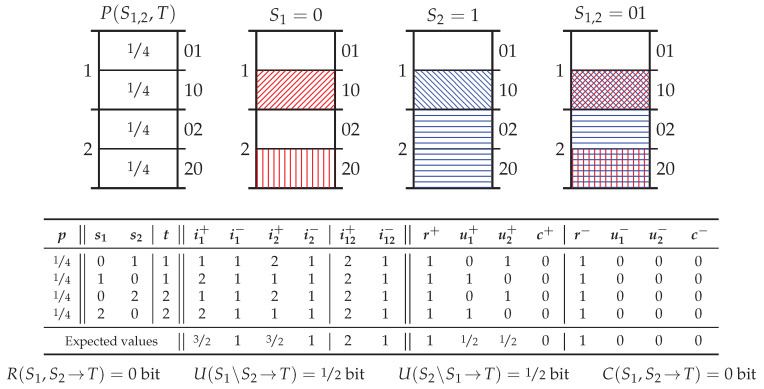
Example PwUnq. (**Top**) probability mass diagrams for the realisation $\left(S_{1}=0,S_{2}=1,T=1\right)$; (**Middle**) For each realisation, the pointwise partial information decomposition (PPID) using specificity and ambiguity is evaluated (see Figure 4 for details). Upon recombination as per (21), the PPI decomposition from Table 1 is attained; (**Bottom**) as does the average information—the decomposition does not have the pointwise unique problem.

Figure 5 shows the probability distribution PwUnq introduced in Section 2.2. Recombining the decomposition via (21) yields the pointwise information decomposition proposed in Table 1—unsurprisingly, the explicitly pointwise approach results in a decomposition which does not suffer from the pointwise unique problem of Section 2.2.

In each realisation, observing a 0 in either source provides the same balanced informative and misinformative exclusions as in Xor. Observing either a 1 or 2 provides the same misinformative exclusion as observing the 0, but provides a larger informative exclusion than 0. This leaves only the probability mass associated with the event which subsequently occurs remaining (hence why observing a 1 and 2 is fully informative about the target). Information theoretically, in each realisation the predictor events provide 1 bit of redundant pointwise specificity and 1 bit of redundant pointwise ambiguity while the fully informative event additionally provides 1 bit of unique specificity.

### 5.4. Probability Distribution *RdnErr*

**Figure 6:**
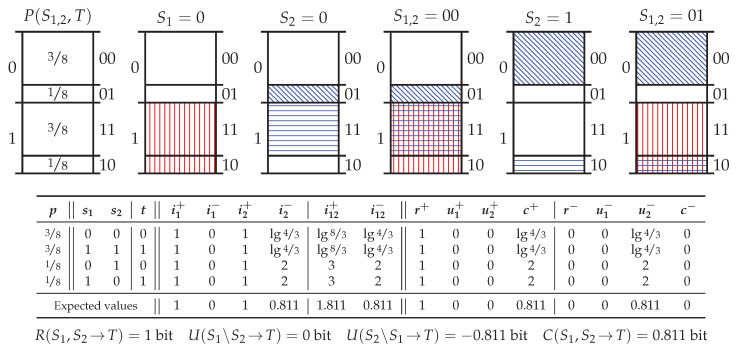
Example RdnErr. (**Top**) probability mass diagrams for the realisations $\left(S_{1}=0,S_{2}=0,T=0\right)$ and $\left(S_{1}=0,S_{2}=1,T=0\right)$; (**Middle**) for each realisation, the PPID using specificity and ambiguity is evaluated (see Figure 4 for details); (**Bottom**) the average PI atoms may be negative as the decomposition does not satisfy local positivity.

Figure 6 shows the probability distribution *redundant-error* (RdnErr) which considers two predictors which are nominally redundant and fully informative about the target, but where one predictor occasionally makes an erroneous prediction. Specifically, Figure 6 shows the decomposition of RdnErr where $S_{2}$ makes an error with a probability ε=14. The important feature to note about this probability distribution is that upon recombining the specificity and ambiguity and taking the expectation over every realisation, the resultant average unique information from $S_{2}$ is $U(S_{2}\backslash S_{1}\to T)$ = −0.811 bit.

On first inspection, the result that the average unique information can be negative may seem problematic; however, it is readily explainable in terms of the operational interpretation of Section 3.3. In RdnErr, a source event always excludes exactly 12 of the total probability mass, thus every realisation contains 1 bit of redundant pointwise specificity. The events of the error-free $S_{1}$ induce only informative exclusions and as such provide 0 bit of pointwise ambiguity in each realisation. In contrast, the events in the error-prone $S_{2}$ always induce a misinformative exclusion, meaning that $S_{2}$ provides unique pointwise ambiguity in every realisation. Since $S_{2}$ never provides unique specificity, the average unique information is negative on average.

Despite the negativity of the average unique information, in is important to observe that $S_{2}$ provides 0.189 bit of information since $S_{2}$ also provides 1 bit of average redundant information. It is not that $S_{2}$ provides negative information on average (as this is not possible); rather it is that not all of the information provided by $S_{2}$ (i.e., the specificity) is “useful” ([42], p. 21). This is in contrast to $S_{1}$ which only provides useful specificity. To summarise, it is the unique ambiguity which distinguishes the information provided by variable $S_{2}$ from $S_{1}$, and hence why $S_{2}$ is deemed to provide negative average unique information. This form of uniqueness can only be distinguished by allowing the average unique information to be negative. This of course, requires abandoning the local positivity as a required property, as per Theorem 4. Few of the existing measures in the PID literature consider dropping this requirement as negative information quantities are typically regarded as being “unfortunate” ([43], p. 49). However, in the context of the pointwise mutual information, negative information values are readily interpretable as being misinformative values. Despite this, the average information from each predictor must be non-negative; however, it may be that what distinguishes one predictor from another are precisely the misinformative predictor events, meaning that the unique information is in actual fact, unique misinformation. Forgoing local positivity makes the PPID using specificity and ambiguity novel (the other exception in this regard is Ince [18] who was first to consider allowing negative average unique information.)

### 5.5. Probability Distribution *Tbc*

**Figure 7:**
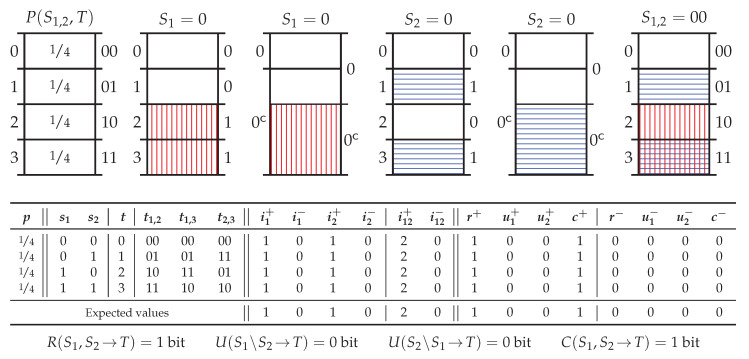
Example Tbc. (**Top**) the probability mass diagrams for the realisation $\left(S_{1}=0,S_{2}=0,T=00\right)$; (**Middle**) for each realisation, the PPID using specificity and ambiguity is evaluated (see Figure 4); (**Bottom**) the decomposition of Xor yields the same result as $I_{\min}$.

Figure 7 shows the probability distribution *two-bit-copy* (Tbc) which considers two independently distributed binary predictor variables $S_{1}$ and $S_{2}$, and a target variable *T* consisting of a separate elementary event for each joint event $S_{1,2}$. There are several important points to note about the decomposition of Tbc. Firstly, due to the symmetry in the probability distribution, each realisation will have the same pointwise decomposition. Secondly, due to the construction of the target, there is an isomorphism (Again, isomorphism should be taken to mean isomorphic probability spaces, e.g., [37], p. 27 or [38], p. 4) between P(T) and $P(S_{1},S_{2})$, and hence the pointwise ambiguity provided by any (individual or joint) predictor event is 0 bit (since given *t*, one knows $s_{1}$ and $s_{2}$). Thirdly, the individual predictor events $s_{1}$ and $s_{2}$ each exclude 12 of the total probability mass in P(T) and so each provide 1 bit of pointwise specificity; thus, by (23), there is 1 bit of redundant pointwise specificity in each realisation. Fourthly, the joint predictor event $s_{1,2}$ excludes 34 of the total probability mass, providing 2 bit of pointwise specificity; hence, by (18), each joint realisation provides 1 bit of pointwise complementary specificity in addition to the 1 bit of redundant pointwise specificity. Finally, putting this together via (22), Tbc consists of 1 bit of average redundant information and 1 bit of average complementary information.

Although “surprising” ([5], p. 268), according to the operational interpretation adopted in Section 3.3, two independently distributed predictor variables can share redundant information. That is, since the exclusions induced by $s_{1}$ and $s_{2}$ are the same with respect to the two-event partition $T^{t}$, the information associated with these exclusions is regarded as being the same. Indeed, this probability distribution highlights the significance of specific reference to the two-event partition in Section 3.3 and Axiom 4. (This can be seen in the probability mass diagram in Figure 7, where the events $S_{1}=0$ and $S_{2}=0$ exclude different elementary target events within the complementary event $0^{c}$ and yet are considered to be the same exclusion with respect to the two-event partition $T^{0}$.) That these exclusions should be regarded as being the same is discussed further in Appendix A. Now however, there is a need to discuss Tbc in terms of Theorem 5 (Target Chain Rule).

Tbc was first considered as a “mechanism” ([6], p. 3) where “the wires don’t even touch” ([12], p. 167), which merely copies or concatenates $S_{1}$ and $S_{2}$ into a composite target variable $T_{1,2}=\left(T_{1},T_{2}\right)$ where $T_{1}=S_{1}$ and $T_{2}=S_{2}$. However, using causal mechanisms as a guiding intuition is dubious since different mechanisms can yield isomorphic probability distributions ([44], and references therein). In particular, consider two mechanisms which generate the composite target variables $T_{1,3}=\left(T_{1},T_{3}\right)$ and $T_{2,3}=\left(T_{2},T_{3}\right)$ where $T_{3}=S_{1}\;\mathrm{XOR}\;S_{2}$. As can be seen in Figure 7, both of these mechanisms generate the same (isomorphic) probability distribution $P(S_{1},S_{2},T)$ as the mechanism generating $T_{1,2}$. If an information decomposition is to depend only on the probability distribution $P(S_{1},S_{2},T)$, and no other semantic details such as labelling, then all three mechanisms must yield the same information decomposition—this is not clear from the mechanistic intuition.

Although the decomposition of the various composite target variables must be the same, there is no requirement that the three systems must yield the same decomposition when analysed in terms of the individual components of the composite target variables. Nonetheless, there ought to be a consistency between the decomposition of the composite target variables and the decomposition of the component target variables—i.e., there should be a target chain rule. As shown in Theorem 5, the measures $r_{\min}$ and $R_{\min}$ satisfy the target chain rule, whereas $I_{\min}$, $\tilde{UI}$, $I_{\mathrm{red}}$ and $S_{\mathrm{VK}}$ do not [5,7]. Failing to satisfy the target chain rule can lead to inconsistencies between the composite and component decompositions, depending on the order in which one considers decomposing the information (this is discussed further in Appendix A.3). In particular, Table 2 shows how $\tilde{UI}$, $I_{\mathrm{red}}$ and $S_{\mathrm{VK}}$ all provide the same inconsistent decomposition for Tbc when considered in terms of the composite target variable $T_{1,3}$. In contrast, $R_{\min}$ produces a consistent decomposition of $T_{1,3}$. Finally, based on the above isomorphism, consider the following (the proof is deferred to Appendix B.3).

**Theorem** **6.** The target chain rule, identity property and local positivity, cannot be simultaneously satisfied.

### 5.6. Summary of Key Properties

The following are the key properties of the PPID using the specificity and ambiguity. Property 1 follows directly from the Definitions 1 and 2. Property 2 follows from Theorems 3 and 4. Property 3 follows from the probability distribution Tbc in Section 5.5. Property 4 was discussed in Section 4.2. Property 5 is proved in Theorem 5.

**Property** **1.** When considering the redundancy between the source events $a_{1},\ldots ,a_{k}$, at least one source event $a_{i}$ will provide zero unique specificity, and at least one source event $a_{j}$ will provide zero unique ambiguity. The events $a_{i}$ and $a_{j}$ are not necessarily the same source event.

**Property** **2.** The atoms of partial specificity and partial ambiguity satisfy local positivity, $\pi^{\pm }\geq 0$. However, upon recombination and averaging, the atoms of partial information do not satisfy local positivity, Π≥0.

**Property** **3.** The decomposition does not satisfy the identity property.

**Property** **4.** The decomposition does not satisfy the target monotonicity property.

**Property** **5.** The decomposition satisfies the target chain rule.

## 6. Conclusions

The partial information decomposition of Williams and Beer [1,2] provided an intriguing framework for the decomposition of multivariate information. However, it was not long before “serious flaws” ([11], p. 2163) were identified. Firstly, the measure of redundant information $I_{\min}$ failed to distinguish between whether predictor variables provide the same information or merely the same amount of information. Secondly, $I_{\min}$ fails to satisfy the target chain rule, despite this addativity being one of the defining characteristics of information. Notwithstanding the problems, the axiomatic derivation of the redundancy lattice was too elegant to be abandoned and hence several alternate measures were proposed, i.e., $I_{\mathrm{red}}$, $\tilde{UI}$ and $S_{\mathrm{VK}}$ [6,11,12]. Nevertheless, as these measures all satisfy the identity property, they cannot produce a non-negative decomposition for an arbitrary number of variables [13]. Furthermore, none of these measures satisfy the target chain rule meaning they produce inconsistent decompositions for multiple target variables. Finally, in spite of satisfying the identity property (which many consider to be desirable), these measures still fail to identify when variables provide the same information, as exemplified by the pointwise unique problem presented in Section 2.

This paper took the axiomatic derivation of the redundancy lattice from PID and applied it to the unsigned entropic components of the pointwise mutual information. This yielded two separate redundancy lattices—the specificity and the ambiguity lattices. Then based upon an operational interpretation of redundancy, measures of pointwise redundant specificity $r_{\min}^{+}$ and pointwise redundant ambiguity $r_{\min}^{-}$ were defined. Together with specificity and ambiguity lattices, these measures were used to decompose multivariate information for an arbitrary number of variables. Crucially, upon recombination, the measure $r_{\min}$ satisfies the target chain rule. Furthermore, when applied to PwUnq, these measures do not result in the pointwise unique problem. In our opinion, this demonstrates that the decomposition is indeed correctly identifying redundant information. However, others will likely disagree with this point given that the measure of redundancy does not satisfy the identity property. According to the identity property, independent variables can never provide the same information. In contrast, according to the operational interpretation adopted in this paper, independent variables can provide the same information if they happen to provide the same exclusions with respect to the two-event target distribution. In any case, the proof of Theorem 6 and the subsequent discussion in Appendix B.3, highlights the difficulties that the identity property introduces when considering the information provided about events in separate target variables. (See further discussion in Appendix A.3).

Our future work with this decomposition will be both theoretical and empirical. Regarding future theoretical work, given that the aim of information decomposition is to derive measures pertaining to sets of random variables, it would be worthwhile to derive the information decomposition from first principles in terms of measure theory. Indeed, such an approach would surely eliminate the semantic arguments (about what it means for information to unique, redundant or complementary), which currently plague the problem domain. Furthermore, this would certainly be a worthwhile exercise before attempting to generalise the information decomposition to continuous random variables. Regarding future empirical work, there are many rich data sets which could be decomposed using this decomposition including financial time-series and neural recordings, e.g., [28,33,34].

## Figures and Tables

**Table 1:** Example PwUnq. For each realisation, the pointwise mutual information provided by each individual and joint predictor events, about the target event has been evaluated. Note that one predictor event always provides full information about the target while the other provides zero information. Based on the this, it is assumed that there must be zero redundant information. The pointwise partial information (PPI) atoms are then calculated via (3).

*p*	$s_{1}$	$s_{2}$	*t*	$i(s_{1};t)$	$i(s_{2};t)$	$i(s_{1,2};t)$	$u(s_{1}\backslash s_{2}\to t)$	$u(s_{2}\backslash s_{1}\to t)$	$r(s_{1},s_{2}\to t)$	$c(s_{1},s_{2}\to t)$
14	0	1	1	0	1	1	0	1	0	0
14	1	0	1	1	0	1	1	0	0	0
14	0	2	2	0	1	1	0	1	0	0
14	2	0	2	1	0	1	1	0	0	0
Expected values				12	12	1	12	12	0	0

**Table 2:** Shows the decomposition of the quantities in the first row induced by the measures in the first column. For consistency, the decomposition of $I(S_{1,2};T_{1,3})$ should equal both the sum of the decomposition of $I(S_{1,2};T_{1})$ and $I(S_{1,2};T_{3}|T_{1})$, and the sum of the decomposition of $I(S_{1,2};T_{3})$ and $I(S_{1,2};T_{1}|3)$. Note that the decomposition induced by $\tilde{UI}$, $I_{\mathrm{red}}$ and $S_{\mathrm{VK}}$ are not consistent. In contrast, $R_{\min}$ is consistent due to Theorem 5.

	$I(S_{1,2};T_{1,3})$	$I(S_{1,2};T_{1})$	$I(S_{1,2};T_{3}|T_{1})$	$I(S_{1,2};T_{3})$	$I(S_{1,2};T_{1}|T_{3})$
$\begin{array}{c} \tilde{UI},\;I_{\mathrm{red}},\\ S_{\mathrm{VK}}\; \end{array}$	$\begin{array}{cc} U(S_{1}\backslash S_{2}\to T_{1,3}) & =1\\ U(S_{2}\backslash S_{1}\to T_{1,3}) & =1 \end{array}$	$U(S_{1}\backslash S_{2}\to T_{1})=1$	$U(S_{2}\backslash S_{1}\to T_{3}|T_{1})=1$	$C(S_{1},S_{2}\to T_{3})=1$	$R(S_{1},S_{2}\to T_{1}|T_{3})=1$
$R_{\min}$	$\begin{array}{cc} R(S_{1},S_{2}\to T_{1,3}) & =1\\ C(S_{1},S_{2}\to T_{1,3}) & =1 \end{array}$	$\begin{array}{cc} U(S_{2}\backslash S_{1}\to T_{1}) & =-1\\ R(S_{1},S_{2}\to T_{1}) & =1\\ C(S_{1},S_{2}\to T_{1}) & =1 \end{array}$	$U(S_{2}\backslash S_{1}\to T_{3}|T_{1})=1$	$C(S_{1},S_{2}\to T_{3})=1$	$R(S_{1},S_{2}\to T_{1}|T_{3})=1$

**Figure A1:**
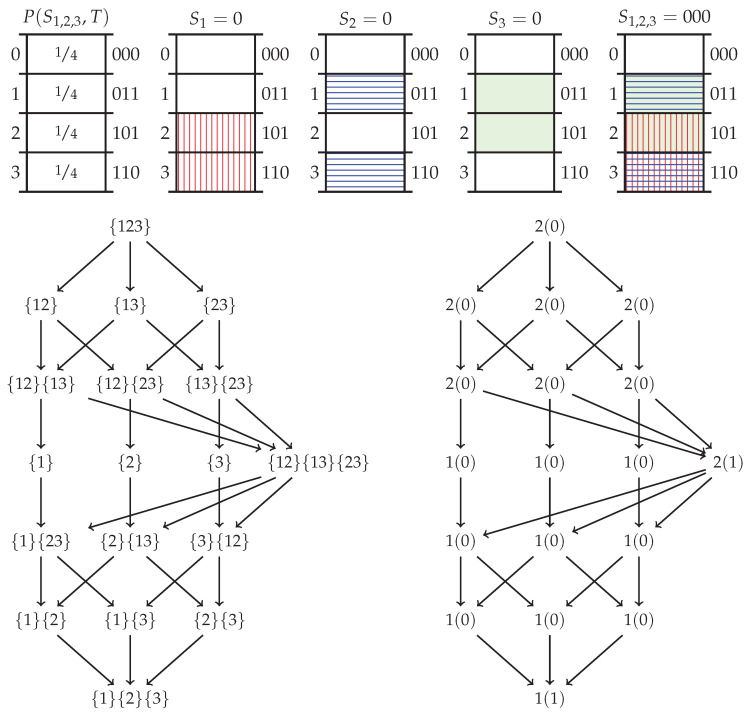
Example Tbep. (**Top**) probability mass diagram for realisation $\left(S_{1}=0,S_{2}=0,S_{3}=0,T=000\right)$; (**Bottom left**) With three predictors, it is convenient to represent to decomposition diagrammatically. This is especially true Tbep as one only needs to consider the specificity lattice for one realisation; (**Bottom right**) The specificity lattice for the realisation $\left(S_{1}=0,S_{2}=0,S_{3}=0,T=000\right)$. For each source event the left value corresponds to the value of $i_{\cap }^{+}$, evaluated using $r_{\min}^{+}$, while the right value (surrounded by parenthesis) corresponds to the partial information $\pi^{+}$.

**Figure A2:**
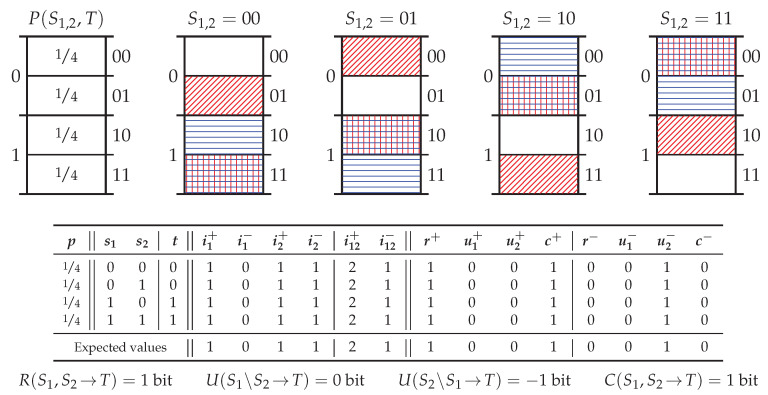
Example Unq. (**Top**) the probability mass diagrams for every single possible realisation; (**Middle**) for each realisation, the PPID using specificity and ambiguity is evaluated (see Figure 4); (**Bottom**) the atoms of (average) partial infromation obtained through recombination of the averages.

**Figure A3:**
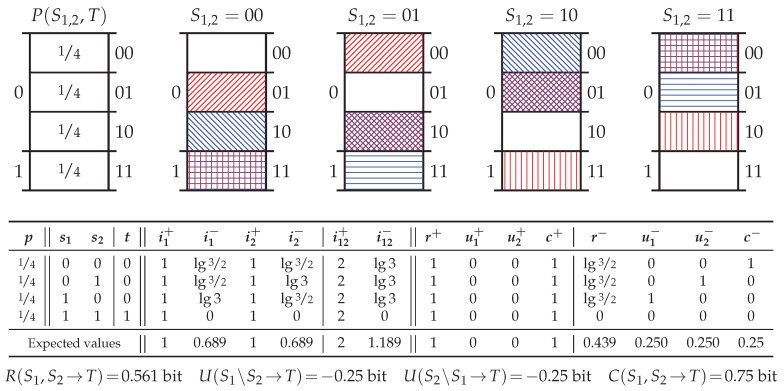
Example And. (**Top**) the probability mass diagrams for every single possible realisation; (**Middle**) for each realisation, the PPID using specificity and ambiguity is evaluated (see Figure 4); (**Bottom**) the atoms of (average) partial infromation obtained through recombination of the averages.

## Appendix A. Kelly Gambling, Axiom 4, and Tbc

In Section 3.3, it was argued that the information provided by a set of predictor events $s_{1},\ldots ,s_{k}$ about a target event *t* is the same information if each source event induces the same exclusions with respect to the two-event partition $T^{t}=\left\{t,t^{c}\right\}$. This was based on the fact that pointwise mutual information does not depend on the apportionment of the exclusions across the set of events which did not occur $t^{c}$. It was argued that since the pointwise mutual information is independent of these differences, the redundant mutual information should also be independent of these differences. This requirement was then integrated into the operational interpretation of Section 3.3 and was later enshrined in the form of Axiom 4. This appendix aims to justify this operational interpretation and argue why redundant information in Tbc is not “unreasonably large” ([5], p. 269).

### Appendix A.1. Pointwise Side Information and the Kelly Criterion

Consider a set of horses T running in a race which can be considered a random variable *T* with distribution P(T). Say that for each t∈T a bookmaker offers odds of o(t)-for-1, i.e., the bookmaker will pay out o(t) dollars on a $1 bet if the horse *t* wins. Furthermore, say that there is no track take as ∑t∈T1o(t)=1, and these odds are *fair*, i.e., o(t)=1p(t) for all t∈T [40]. Let b(T) be the fraction of a gambler’s capital bet on each horse t∈T and assume that the gambler stakes all of their capital on the race, i.e., $\sum_{t\in T}b\left(t\right)=1$.

Now consider an i.i.d. series of these races $T_{1},T_{2},\ldots$ such that $P\left(T_{k}\right)=P\left(T\right)$ for all k∈N and let $t_{k}\in T$ represent the winner of the *k*-th race. Say that the bookmaker offers the same odds on each race and the gambler bets their entire capital on each race. The gambler’s capital after *m* races $D_{m}$ is a random variable which depends on two factors per race: the amount the gambler staked on each race winner $t_{k}$, and the odds offered on each winner $t_{k}$. That is,

(A1) $$D_{m}=\prod_{k=1}^{m}b\left(t_{k}\right)\;o\left(t_{k}\right),$$

where monetary units $ have been chosen such that $D_{0}$ = $1. The gambler’s wealth grows (or shrinks) exponentially, i.e.,

(A2) $$D_{m}=2^{m\;W(b,T)}$$

where

(A3) $$W\left(b,T\right)=\frac{1}{m}\log D_{m}=\frac{1}{m}\sum_{k=1}^{m}\log b\left(t_{k}\right)\;o\left(t_{k}\right)=E\left[\log b\left(t_{k}\right)\;o\left(t_{k}\right)\right]$$

is the doubling rate of the gambler’s wealth using a betting strategy b(T). Here, the last equality is by the weak law of large numbers for large *m*.

Any reasonable gambler would aim to use an optimal strategy $b^{*}\left(T\right)$ which maximises the doubling rate W(b,T). Kelly [40,43] proved that the optimal doubling rate is given by

(A4) $$W^{*}\left(T\right)=\max_{b}W\left(b,T\right)=E\left[\log b^{*}\left(t_{k}\right)\;o\left(t_{k}\right)\right]$$

and is achieved by using the proportional gambling scheme $b^{*}\left(T\right)=P\left(T\right)$. When the race $T_{k}$ occurs and the horse $t_{k}$ wins, the gambler will receive a payout of $b^{*}\left(t^{k}\right)\;o\left(t^{k}\right)$ = $1, i.e., the gambler receives their stake back regardless of the outcome. In the face of fair odds, the proportional Kelly betting scheme is the optimal strategy—non-terminating repeated betting with any other strategy will result in losses.

Now consider a gambler with access to a private wire *S* which provides (potentially useful) side information about the upcoming race. Say that these messages are selected from the set S, and that the gambler receives the message $s_{k}$ before the race $T_{k}$. Kelly [40,43] showed that the optimal doubling rate in the presence of this side information is given by

(A5) $$W^{*}\left(T|S\right)=\max_{b}W\left(b,T|S\right)=E\left[\log b^{*}\left(t_{k}|s_{k}\right)\;o\left(t_{k}\right)\right],$$

and is achieved by using the conditional proportional gambling scheme $b^{*}\left(T|s_{k}\right)=P\left(T|s_{k}\right)$. Both the proportional gambling scheme $b^{*}\left(T\right)$ and the conditional proportional gambling scheme $b^{*}\left(T|S\right)$ are based upon the *Kelly criterion* whereby bets are apportioned according to the best estimation of the outcome available. The financial value of the private wire to a gambler can be ascertained by comparing their doubling rate of the gambler with access to the side wire to that of a gambler with no side information, i.e.,

(A6) $$\begin{array}{cc} \Delta W=W^{*}\left(T|S\right)-W^{*}\left(T\right) & =E\left[\log b^{*}\left(t_{k}|s_{k}\right)\;o\left(t_{k}\right)\right]-E\left[\log b^{*}\left(t_{k}\right)\;o\left(t_{k}\right)\right]\\ & =E\left[i(s_{k};t_{k})\right]=I\left(S;T\right). \end{array}$$

This important result due to Kelly [40] equates the increase in the doubling rate ΔW due to the presence of side information, with the mutual information between the private wire *S* and the horse race *T*. If on average, the gambler receives 1 bit of information from their private wire, then on average the gambler can expect to double their money per race. Furthermore, as one would expect, independent side information does not increase the doubling rate.

With no side information, the Kelly gambler always received their original stake back from the bookmaker. However, this is not true for the Kelly gambler with side information. Although their doubling rate is greater than or equal to that of the gambler with no side information, this is only true *on average*. Before the race $T_{k}$, the gambler receives the private wire message $s_{k}$ and then, the horse $t_{k}$ wins the race. From (A6), one can see that the return $\Delta w_{k}$ for the *k*-th race is given by the pointwise mutual information,

(A7) $$\Delta w=i(s_{k};t_{k}).$$

Hence, just like the pointwise mutual information, the per race return can be positive or negative: if it is positive, the gambler will make a profit; if it is negative, the gambler will sustain a loss. Despite the potential for pointwise loses, the average return (i.e., the doubling rate) is, just like the average mutual information, non-negative—and indeed, is optimal. Furthermore, while a Kelly gambler with side information can lose money on any single race, they can never actually go bust. The Kelly gambler with side information *s* still hedges their risk by placing bets on all horses with a non-zero probability of winning according to their side information, i.e., according to $P(T|s_{k})$. The only reason they would fail to place a bet on a horse is if their side information completely precludes any possibility of that horse winning. That is, a Kelly gambler with side information will never fall foul of gambler’s ruin.

### Appendix A.2. Justification of Axiom 4 and Redundant Information in *Tbc*

Consider Tbc semantically described in terms of a horse race. That is, consider a four horse race *T* where each horse has an equiprobable chance of winning, and consider the binary variables $T_{1}$, $T_{2}$, and $T_{3}$ which represent the following, respectively: the colour of the horse, black 0 or white 1; the sex of the jockey, female 0 or male 1; and the colour of the jockey’s jersey, red 0 or green 1. Say that the four horses have the following attributes:

Horse 0 is a black horse $T_{1}=0$, ridden by a female jockey $T_{2}=0$, who is wearing a red jersey $T_{3}=0$.
Horse 1 is a black horse $T_{1}=0$, ridden by a male jockey $T_{2}=1$, who is wearing a green jersey $T_{3}=1$.
Horse 2 is a white horse $T_{1}=1$, ridden by a female jockey $T_{2}=0$, who is wearing a green jersey $T_{3}=1$.
Horse 3 is a white horse $T_{1}=1$, ridden by a male jockey $T_{2}=1$, who is wearing a red jersey $T_{3}=0$.

There are two important points to note. Firstly, the horses in the race *T* could also be uniquely described in terms of the composite binary variables $T_{1,2}$, $T_{1,3}$ or $T_{2,3}$. Secondly, if one knows $T_{1}$ and $T_{2}$ then one knows $T_{3}$ (which can be represented by the relationship $T_{3}=T_{1}\;\mathrm{XOR}\;T_{2}$). Finally, consider private wires $S_{1}$ and $S_{2}$ which *independently* provide the colour of the horse and the colour of the jockey’s jersey (respectively) before the upcoming race, i.e., $S_{1}=T_{1}$ and $S_{2}=T_{2}$.

Now say a bookmaker offers fair odds of 4-for-1 on each horse in the race *T*. Consider two gamblers who each have access to one of $S_{1}$ and $S_{2}$. Before each race, the two gamblers receive their respective private wire messages and place their bets according to the Kelly strategy. This means that each gambler lays half of their, say $1, stake on each of their two respective non-excluded horses: unknowingly, both of the gamblers have placed a bet on the soon-to-be race winner, and each gambler has placed a distinct bet on one of the two soon-to-be losers. The only horse neither has bet upon is also a soon-to-be loser. (See [5] for a related description of Tbc in term of the game-theoretic notions of shared and common knowledge). After the race, the bookmaker pays out $2 dollars to each gambler: both have doubled their money. This happens because both of the gamblers had one bit of 1 bit of information about the race, i.e., pointwise mutual information. In particular, both gamblers improved their probability of predicting the eventual race winner. It did not matter, in any way, that the gamblers had each laid distinct bets on one of the three eventual race losers. The fact that they laid different bets on the horses which did not win, made no difference to their winnings. The apportionment of the exclusions across the set of events which did not occur, makes no difference to the pointwise mutual information. With respect to what occurred (i.e., with respect to which horse won), the fact the that they excluded different losers is only semantic. When it came to predicting the would-be-winner, both gamblers had the same predictive power; they both had the same freedom of choice with regards to selecting what would turn out to be the eventual race winner—they had the same information. It is for this reason that this information should be regarded as redundant information, regardless of the independence of the information sources. Hence, the introduction of both the operational interpretation of redundancy in Section 3.3 and Axiom 4 in Section 4.2.

Now consider a third gambler who has access to both private wires $S_{1}$ and $S_{2}$, i.e., $S_{1,2}$. Before the race, this gambler receives both private wire messages which, in total, precludes three of the horses from winning. This gambler then places the entirety of their $1 stake on the remaining horse which is sure to win. After the race, the bookmaker pays out $4: this gambler has quadrupled their money as they had 2 bit of pointwise mutual information about the race. Having both private wire messages simultaneously gave this gambler a 1 bit informational edge over the two gamblers with access to a single side wire. While each of the singleton gamblers had 1 bit of independent information, the only way one could profit from the independence of this information is by having both pieces of information simultaneously—this makes this 1 bit of information complementary. Although this may seem “palpably strange” ([12], p. 167), it is not so strange when from the following perspective: the only way to exploit two pieces of independent information is by having both pieces together simultaneously.

### Appendix A.3. Accumulator Betting and the Target Chain Rule

Say that in addition to the 4-for-1 odds offered on the race *T*, the bookmaker also offers fair odds of 2-for-1 on each of the binary variables $T_{1}$, $T_{2}$ and $T_{3}$. Now, in addition to being able to directly gamble on the race *T*, one could indirectly gamble on *T* by placing a so-called *accumulator* bet on any pair of $T_{1}$, $T_{2}$ and $T_{3}$. An accumulator is a series of chained bets whereby any return from one bet is automatically staked on the next bet; if any bet in the chain is lost then the entire chain is lost. For example, a gambler could place 4-for-1 bet on horse 0 by placing the following accumulator bet: a 2-for1 bet on a black horse winning that chains into a 2-for-1 bet on the winning jockey being female (or equivalently, vice versa). In effect, these accumulators enable a gambler to bet on *T* by instead placing a chained bet on the independent component variables within the (equivalent) joint variables $T_{1,2}$, $T_{1,3}$ and $T_{2,3}$. Now consider again the three gamblers from the prior section, i.e., the two gamblers who each have a private wire $S_{1}$ and $S_{2}$, and the third gamble who has access to $S_{1,2}$. Say that they must each place a, say $1, accumulator bet on $T_{1,3}$—what should each gambler do according to the Kelly criterion?

For the sake of clarity, consider only the realisation where the horse T=0 subsequently wins (due to the symmetry, the analysis is equivalent for all realisations). First consider the accumulator whereby the gamblers first bet on the colour of the winning horse $T_{1}$, which chains into a bet on the colour of the winning jockey’s jersey $T_{3}$. Suppose that the private wire $S_{1}$ communicates that the winning horse will be black, while the private wire $S_{2}$ communicates that the winning horse will be ridden by a female jockey, i.e., $S_{1}=0$ and $S_{2}=0$. Following to the Kelly strategy, the gambler with access to $S_{1}=0$ takes out two $0.5 accumulator bets. Both of these accumulators feature the same initial bet on the winning horse being black since $T_{1}=S_{1}=0$. Hence both bets return $1 each which become the stake on the next bet in each accumulator. This gambler knows nothing about the colour of the jockey’s jersey $T_{3}$. As such, one accumulator chains into a bet on the winning jersey being red $T_{3}=0$, while the other chains into a bet on it being green $T_{3}=1$. When the horse T=0 wins, the stake bet on the green jersey is lost while bet on red jersey pays out $2. This gambler had 1 bit of side information and so doubled their money. Now consider the gambler with private wire $S_{2}$, who knows nothing about $T_{1}$ or $T_{3}$ individually. Nonetheless, this gambler knows that the winner must be a female jockey $T_{2}=0$. As such, this gambler knows that if a black horse $T_{1}=0$ wins then its jockey must be wearing a red jersey $T_{3}=0$, or if a white horse $T_{1}=0$ wins then its jockey must be wearing a green jersey $T_{3}=1$ (since $T_{3}=T_{1}\;\mathrm{XOR}\;T_{2}$). Thus this gambler can also utilise the Kelly strategy to place the following two $0.5 accumulator bets: the first accumulator bets on the winning horse being black $T_{1}=0$ and then chains into a bet on the winner’s jersey being red $T_{3}=0$, while the second accumulator bets on the winning horse being white $T_{1}=1$ and then chains into a bet on the winner’s jersey being green $T_{3}=1$. When the horse T=0 wins, the first accumulator pays out $2, while the second accumulator is be lost. Hence, this gambler also doubles their money and so also had 1 bit of side information. Finally, consider the gambler with access to both private wires $S_{1,3}$, who can place an accumulator on the black horse $T_{1}=0$ winning chaining into a bet on the winning jockey wearing red $T_{3}=0$. This gambler can quadruple their stake, and so must possess 2 bit of side information.

Each of the three gamblers have the same final return regardless of whether the gamblers are betting on the variable *T*, or placing accumulator bets on the variables $T_{1,2}$, $T_{1,3}$ or $T_{2,3}$. However, the paths to the final result differs between the gamblers, reflecting the difference between the information the each gambler had about the sub-variables $T_{1}$, $T_{2}$ or $T_{3}$. Given the result of Kelly [40], the proposed information decomposition should reflect these differences, but yet still arrive at the same result—in other words, the information decomposition should satisfy a target chain rule. This is clear if the Kelly interpretation of information is to remain as a “duality” ([43], p. 159) in information theory.

## Appendix B. Supporting Proofs and Further Details

This appendix contains many of the important theorems and proofs relating to PPID using specificity and ambiguity.

### Appendix B.1. Deriving the Specificity and Ambiguity Lattices from Axioms 1–4

The following section is based directly on the original work of Williams and Beer [1,2]. The difference is that we now consider sources events $a_{i}$ rather than sources $A_{i}$.

**Proposition** **A1.** *Both $i_{\cap }^{+}$ and $i_{\cap }^{-}$ are non-negative.*

**Proof.** Since $\emptyset \subseteq a_{i}$ for any $a_{i}$, Axioms 2 and 3 imply

(A8) $$\begin{array}{ccccc} i_{\cap }^{+}\left(a_{1},\cdots ,a_{k}\to t\right) & \geq i_{\cap }^{+}\left(a_{1},\cdots ,a_{k},\emptyset \to t\right) & =i_{\cap }^{+}\left(\emptyset \to t\right) & =h(\emptyset ) & =0, \end{array}$$

(A9) $$\begin{array}{ccccc} i_{\cap }^{-}\left(a_{1},\cdots ,a_{k}\to t\right) & \geq i_{\cap }^{-}\left(a_{1},\cdots ,a_{k},\emptyset \to t\right) & =i_{\cap }^{-}\left(\emptyset \to t\right) & =h(\emptyset |t) & =0. \end{array}$$

Hence, both $i_{\cap }^{+}\left(a_{1},\cdots ,a_{k}\to t\right)$ and $i_{\cap }^{-}\left(a_{1},\cdots ,a_{k}\to t\right)$ are non-negative. ☐

**Proposition** **A2.** *Both $i_{\cap }^{+}$ and $i_{\cap }^{-}$ are bounded from above by the specificity and the ambiguity from any single source event, respectively.*

**Proof.** For any single source $a_{i}$, Axioms 2 and 3 yield

(A10) $$\begin{array}{cccc} h(a_{i}) & =i_{\cap }^{+}\left(a_{i}\to t\right) & =i_{\cap }^{+}\left(a_{i},a_{i}\to t\right) & \geq i_{\cap }^{+}\left(a_{i},\cdots \to t\right), \end{array}$$

(A11) $$\begin{array}{cccc} h(a_{i}|t) & =i_{\cap }^{-}\left(a_{i}\to t\right) & =i_{\cap }^{-}\left(a_{i},a_{i}\to t\right) & \geq i_{\cap }^{+}\left(a_{i},\cdots \to t\right), \end{array}$$

as required. ☐

In keeping with Williams and Beer’s approach [1,2], consider all of the distinct ways in which a collection of source events $a=\{a_{1},\cdots ,a_{k}\}$ could contribute redundant information. Thus far we have assumed that the redundancy measure can be applied to any collection of source events, i.e., $P_{1}\left(a\right)$ where $P_{1}$ denotes the power set with the empty set removed. Recall that the sources events are themselves collections of predictor events, i.e., $P_{1}\left(s\right)$. That is, we can apply both $i_{\cap }^{+}$ and $i_{\cap }^{-}$ to elements of $P_{1}\left(P_{1}\left(s\right)\right)$. However, this can be greatly reduced using Axiom 2 which states that if $a_{i}\subseteq a_{j}$, then

(A12) $$\begin{array}{cc} i_{\cap }^{+}\left(a_{j},a_{i},\ldots \to t\right) & =i_{\cap }^{+}\left(a_{i},\ldots \to t\right), \end{array}$$

(A13) $$\begin{array}{cc} i_{\cap }^{-}\left(a_{j},a_{i},\ldots \to t\right) & =i_{\cap }^{-}\left(a_{i},\ldots \to t\right). \end{array}$$

Hence, one need only consider the collection of source events such that no source event is a superset of any other in order,

(A14) $$A\left(s\right)=\left\{\alpha \in P_{1}\left(P_{1}\left(s\right)\right)\;|\;\forall \;a_{i},\;a_{j}\in \alpha ,\;a_{i}⊄a_{j}\right\}.$$

This collection A(s) captures all the distinct ways in the source events could provide redundant information.

As per Williams and Beer’s PID, this set of source events A(s) is structured. Consider two sets of source events α,β∈A(s). If for every source event b∈β there exists a source event a∈α such that a⊆b, then all of the redundant specificity and ambiguity shared by b∈β must include any redundant specificity and ambiguity shared by a∈α. Hence, a partial order ⪯ can be defined over the elements of the domain A(s) such that any collection of predictors event coalitions precedes another if and only if the latter provides any information the former provides,

(A15) $$\forall \alpha ,\beta \in A\left(s\right),\left(\alpha ⪯\beta \Leftrightarrow \forall \;b\in \beta ,\;\exists \;a\in \alpha \;|\;a\subseteq b\right).$$

Applying this partial ordering to the elements of the domain A(s) produces a lattice which has the same structure as the redundancy lattice from PID, i.e., the structure of the sources events here is the same as the structure of the sources in PID. (Figure 3 depicts this structure for the case of 2 and 3 predictor variables.) Applying $i_{\cap }^{+}$ to these sources events yields a *specificity lattice* while applying $i_{\cap }^{-}$ yields an *ambiguity lattice*.

Similar to $I_{\cap }$ in PID, the redundancy measures $i_{\cap }^{+}$ or $i_{\cap }^{-}$ can be thought of as a cumulative information functions which integrate the specificity or ambiguity uniquely contributed by each node as one moves up each lattice. In order in evaluate the unique contribution of specificity and ambiguity from each node in the lattice, consider the Möbius inverse [45,46] of $i_{\cap }^{+}$ and $i_{\cap }^{-}$. That is, the specificity and ambiguity of a node α is given by

(A16) $$i_{\cap }^{\pm }\left(\alpha \to t\right)=\sum_{\beta ⪯\alpha }i_{\cap }^{\pm }\left(\beta \to t\right)\;\forall \;\alpha ,\;\beta \in A\left(s\right).$$

Thus the unique contributions of *partial specificity* $i_{\partial }^{+}$ and *partial ambiguity* $i_{\partial }^{-}$ from each node can be calculated recursively from the bottom-up, i.e.,

(A17) $$i_{\partial }^{\pm }\left(\alpha \to t\right)=i_{\cap }^{\pm }\left(\alpha \to t\right)-\sum_{\beta ≺\alpha }i_{\partial }^{\pm }\left(\beta \to t\right).$$

**Theorem** **A1.** *Based on the principle of inclusion-exclusion, we have the following closed-from expression for the partial specificity and partial ambiguity,*

(A18) $$i_{\partial }^{\pm }\left(\alpha \to t\right)=i_{\cap }^{\pm }\left(\alpha \to t\right)\;-\sum_{\emptyset \neq \gamma \subseteq \alpha^{-}}{\left(-1\right)}^{|\gamma |-1}\;i_{\cap }^{\pm }\left(⋀\gamma \to t\right)$$

**Proof.** For B⊆A(s), define the sub-addative function $f^{\pm }\left(B\right)=\sum_{\beta \in B}=i^{\pm }\left(\beta \to t\right)$. From (A16), we get that $i_{\cap }^{\pm }\left(\alpha \to t\right)=f^{\pm }\left(↓\alpha \right)$ and

(A19) $$i_{\partial }^{\pm }\left(\alpha \to t\right)=f^{\pm }\left(↓\alpha \right)-f^{\pm }\left(\dot{↓}\alpha \right)=f^{\pm }\left(↓\alpha \right)-f^{\pm }\left(\underset{\beta \in \alpha^{-}}{⋃}↓\beta \right).$$

By the principle of inclusion-exclusion (e.g., see [46], p. 195) we get that

(A20) $$\begin{array}{cc} i_{\partial }^{\pm }\left(\alpha \to t\right) & =f^{\pm }\left(↓\alpha \right)\;-\sum_{\emptyset \neq \gamma \subseteq \alpha^{-}}{\left(-1\right)}^{|\gamma |-1}\;f^{\pm }\left(\underset{\beta \in \gamma }{⋂}\beta \right) \end{array}$$

For any lattice *L* and A⊆L, we have that $\cap_{a\in A}↓a=\;↓\left(⋀A\right)$ (see [47], p. 57) thus

(A21) $$\begin{array}{cc} 1-1i_{\partial }^{\pm }\left(\alpha \to t\right) & =f^{\pm }\left(↓\alpha \right)\;-\sum_{\emptyset \neq \gamma \subseteq \alpha^{-}}{\left(-1\right)}^{|\gamma |-1}\;f^{\pm }\left(⋀\gamma \right)\\ & =f^{\pm }\left(↓\alpha \right)-\sum_{\emptyset \neq \gamma \subseteq \alpha^{-}}{\left(-1\right)}^{|\gamma |-1}\;i^{\pm }\left(⋀\gamma \to t\right) \end{array}$$

as required. ☐

Similarly to PID, the specificity and ambiguity lattices provide a structure for information decomposition—unique evaluation requires a separate definition of redundancy. However, unlike PID (or even PPID), this evaluation requires both a definition of pointwise redundant specificity and pointwise redundant ambiguity.

### Appendix B.2. Redundancy Measures on the Lattices

In Section 4.2, Definitions 1 and 2 provided the require measures. This section will prove some of the key properties of these measures when they are applies to the lattices derived in the previous section. The correspondence with the approach taken by Williams and Beer [1,2] continues in this section. However, sources events $a_{i}$ are used in place of sources $A_{i}$ and the measures $r_{\min}^{\pm }$ are used in place of $I_{\min}$. Note that the basic concepts from lattice theory and the notion used here are the same as found in ([1], Appendix B).

**Theorem** **1.** The definitions of $r_{min}^{+}$ and $r_{min}^{-}$ satisfy Axioms 1–4.

**Proof.** Axioms 1, 3 and 4 follow trivially from the basic properties of the minimum. The main statement of Axiom 2 also immediately follows from the properties of the minimum; however, there is a need to verify the equality condition. As such, consider $a_{k}$ such that $a_{k}⊇a_{i}$ for some $a_{i}\in \left\{a_{1},\ldots ,a_{k-1}\right\}$. From Postulate 4, we have that $h\left(a_{k}\right)\geq h\left(a_{i}\right)$ and hence that $\min_{a_{j}\in \left\{a_{1},\ldots ,a_{k}\right\}}h\left(a_{j}\right)=\min_{a_{j}\in \left\{a_{1},\ldots ,a_{k-1}\right\}}h\left(a_{j}\right)$, as required for $r_{\min}^{+}$. *Mutatis mutandis*, similar follows for $r_{\min}^{-}$. ☐

**Theorem** **2.** The redundancy measures $r_{min}^{+}$ and $r_{min}^{-}$ increase monotonically on the $\langle A(s),⪯\rangle$.

The proof of this theorem will require the following lemma.

**Lemma** **A1.** *The specificity and ambiguity $i^{\pm }\left(a\to t\right)$ are increasing functions on the lattice $\langle P_{1}\left(s\right),\subseteq \rangle$*

**Proof.** Follows trivially from Postulate 4. ☐

**Proof** **of** **Theorem** **2.** Assume there exists α,β∈A(s) such that α≺β and $r_{\min}^{\pm }\left(\beta \to t\right)<r_{\min}^{\pm }\left(\alpha \to t\right)$. By definition, i.e., (23) and (24), there exists b∈β such that $i^{\pm }\left(b\to t\right)<i^{\pm }\left(a\to t\right)$ for all a∈α. Hence, by Lemma A1, there does not exist a∈α such that a⊆b. However, by assumption α≺β and hence there exists a∈α such that a⊆b, which is a contradiction. ☐

**Theorem** **A2.** *When using $r_{min}^{\pm }$ in place of the general redundancy measures $i_{\cap }^{\pm }$, we have the following closed-from expression for the partial specificity $\pi^{+}$ and partial ambiguity $\pi^{-}$,*

(A22) $$\pi^{\pm }\left(\alpha \to t\right)=r_{min}^{\pm }\left(\alpha \to t\right)-\max_{\beta \in \alpha^{-}}\;\min_{b\in \beta }\;i^{\pm }\left(b\to t\right).$$

**Proof.** Let $i_{\cap }^{+}=r_{\min}^{+}$ and $i_{\cap }^{-}=r_{\min}^{-}$ in the general closed form expression for $i_{\partial }^{\pm }$ in Theorem A1,

(A23) $$\pi^{\pm }\left(\alpha \to t\right)=r_{\min}^{\pm }\left(\alpha \to t\right)\;-\sum_{\emptyset \neq \gamma \subseteq \alpha^{-}}{\left(-1\right)}^{|\gamma |-1}\min_{b\in ⋀\gamma }\;i^{\pm }\left(b\to t\right).$$

Since $\alpha \wedge \beta =\underline{\alpha \cup \beta }$ (see [1], Equation (23)), and by Postulate 4, we have that

(A24) $$\pi^{\pm }\left(\alpha \to t\right)=r_{\min}^{\pm }\left(\alpha \to t\right)\;-\sum_{\emptyset \neq \gamma \subseteq \alpha^{-}}{\left(-1\right)}^{|\gamma |-1}\min_{\beta \in \gamma }\;\min_{b\in \beta }\;i^{\pm }\left(b\to t\right).$$

By the maximum-minimums identity (see [48]), we have that, $\max\alpha^{-}=\sum_{\emptyset \neq \gamma \subseteq \alpha^{-}}{\left(-1\right)}^{|\gamma |-1}\;\min\gamma$, and hence

(A25) $$\pi^{\pm }\left(\alpha \to t\right)=r_{\min}^{\pm }\left(\alpha \to t\right)-\max_{\beta \in \alpha^{-}}\;\min_{b\in \beta }\;i^{\pm }\left(\alpha \to t\right).$$

as required. ☐

**Theorem** **3.** The atoms of partial specificity $\pi^{+}$ and partial ambiguity $\pi^{-}$ evaluated using the measures $r_{min}^{+}$ and $r_{min}^{-}$ on the specificity and ambiguity lattices (respectively), are non-negative.

**Proof.** It α=⊥, the $\pi^{\pm }\left(\alpha \to t\right)=r_{\min}^{\pm }\geq 0$ by the non-negativity of entropy. If α≠⊥, assume there exists α∈A(s)\{⊥} such that $\pi^{\pm }\left(\alpha \to t\right)<0$. By Theorem A2,

(A26) $$\pi^{\pm }\left(\alpha \to t\right)=\min_{a\in \alpha }\;i^{\pm }\left(a\to t\right)-\max_{\beta \in \alpha^{-}}\;\min_{b\in \beta }\;i^{\pm }\left(b\to t\right).$$

From this it can be seen that there must exist $\beta \in \alpha^{-}$ such that for all b∈β, we have that $i^{\pm }\left(a\to t\right)<i^{\pm }\left(b\to t\right)$ for some a∈α. By Postulate 4 there does not exist b∈β such that b⊂a. However, since by definition, β≺α there exists b∈β such that b⊂a, which is a contradiction. ☐

**Theorem** **4.** The atoms of partial average information Π evaluated by recombining and averaging $\pi^{\pm }$ are not non-negative.

**Proof.** The proof is by the counter-example using RdnErr. ☐

### Appendix B.3. Target Chain Rule

By using the appropriate conditional probabilities in Definitions 1 and 2, one can easily obtain the conditional pointwise redundant specificity,

(A27) $$r_{\min}^{+}\left(a_{1},\cdots ,a_{k}\to t_{1}|t_{2}\right)=\min_{a_{i}}h\left(a_{i}|t_{2}\right),$$

or the conditional pointwise redundant ambiguity,

(A28) $$r_{\min}^{-}\left(a_{1},\cdots ,a_{k}\to t_{1}|t_{2}\right)=\min_{a_{j}}h\left(a_{j}|t_{1,2}\right).$$

As per (21) these could be recombined, e.g., via (21), to obtain the conditional redundant information,

(A29) $$r_{\min}\left(a_{1},\cdots ,a_{k}\to t_{1}|t_{2}\right)=r_{\min}^{+}\left(a_{1},\cdots ,a_{k}\to t_{1}|t_{2}\right)-r_{\min}^{-}\left(a_{1},\cdots ,a_{k}\to t_{1}|t_{2}\right).$$

The relationship between the regular forms and the conditional forms of the redundant specificity and redundant ambiguity has some important consequences.

**Proposition** **A3.** *The conditional pointwise redundant specificity provided by $a_{1},\cdots ,a_{k}$ about $t_{1}$ given $t_{2}$ is equal to pointwise redundant ambiguity provided by $a_{1},\cdots ,a_{k}$ about $t_{2}$ with the conditioned variable,*

(A30) $$r_{min}^{+}\left(a_{1},\cdots ,a_{k}\to t_{1}|t_{2}\right)=r_{min}^{-}\left(a_{1},\cdots ,a_{k}\to t_{2}\right).$$

**Proof.** By (24) and (A27). ☐

**Proposition** **A4.** *The pointwise redundant specificity provided by $a_{1},\cdots ,a_{k}$ is independent of the target event and even the target variable itself,*

(A31) $$r_{min}^{+}\left(a_{1},\cdots ,a_{k}\to t_{1}\right)=r_{min}^{+}\left(a_{1},\cdots ,a_{k}\to t_{2}\right)\;\forall \;t_{1},t_{2},T_{1},T_{2}.$$

**Proof.** By inspection of (23). ☐

**Proposition** **A5.** *The conditional pointwise redundant ambiguity provided by $a_{1},\cdots ,a_{k}$ about $t_{1}$ given $t_{2}$ is equal to the pointwise redundant ambiguity provided by $a_{1},\cdots ,a_{k}$ about $t_{1,2}$,*

(A32) $$r_{min}^{-}\left(a_{1},\cdots ,a_{k}\to t_{1}|t_{2}\right)=r_{min}^{-}\left(a_{1},\cdots ,a_{k}\to t_{1,2}\right).$$

**Proof.** By (24) and (A28). ☐

Note that specificity itself is not a function of the target event or variable. Hence, all of the target dependency is bound up in the ambiguity. Now consider the following.

**Theorem** **5** (Pointwise Target Chain Rule)**.** *Given the joint target realisation $t_{1,2}$, the pointwise redundant information $r_{min}$ satisfies the following chain rule,*

(25) $$\begin{array}{cc} r_{min}\left(a_{1},\ldots ,a_{k}\to t_{1,2}\right) & =r_{min}\left(a_{1},\ldots ,a_{k}\to t_{1}\right)+r_{min}\left(a_{1},\ldots ,a_{k}\to t_{2}|t_{1}\right),\\ & =r_{min}\left(a_{1},\ldots ,a_{k}\to t_{2}\right)+r_{min}\left(a_{1},\ldots ,a_{k}\to t_{1}|t_{2}\right). \end{array}$$

**Proof.** Starting from $r_{\min}$, by Corollary A4 and Corollary A5 we get that

(A33) $$\begin{array}{cc} r_{\min}\left(a_{1},\ldots ,a_{k}\to t_{1,2}\right) & =r_{\min}^{+}\left(a_{1},\ldots ,a_{k}\to t_{1,2}\right)-r_{\min}^{-}\left(a_{1},\ldots ,a_{k}\to t_{1,2}\right),\\ & =r_{\min}^{+}\left(a_{1},\ldots ,a_{k}\to t_{1}\right)-r_{\min}^{-}\left(a_{1},\ldots ,a_{k}\to t_{2}|t_{1}\right), \end{array}$$

Then, by Corollary A3 we get that

(A34) $$\begin{array}{cc} r_{\min}\left(a_{1},\ldots ,a_{k}\to t_{1,2}\right)= & r_{\min}^{+}\left(a_{1},\ldots ,a_{k}\to t_{1}\right)-r_{\min}^{-}\left(a_{1},\ldots ,a_{k}\to t_{1}\right)\\ & \;+r_{\min}^{-}\left(a_{1},\ldots ,a_{k}\to t_{1}\right)-r_{\min}^{-}\left(a_{1},\ldots ,a_{k}\to t_{2}|t_{1}\right),\\ = & r_{\min}^{+}\left(a_{1},\ldots ,a_{k}\to t_{1}\right)-r_{\min}^{-}\left(a_{1},\ldots ,a_{k}\to t_{1}\right)\\ & \;+r_{\min}^{+}\left(a_{1},\ldots ,a_{k}\to t_{2}|t_{1}\right)-r_{\min}^{-}\left(a_{1},\ldots ,a_{k}\to t_{2}|t_{1}\right),\\ = & r_{\min}\left(a_{1},\ldots ,a_{k}\to t_{1}\right)+r_{\min}\left(a_{1},\ldots ,a_{k}\to t_{2}|t_{1}\right), \end{array}$$

as required for the first equality in (25). *Mutatis mutandis*, we obtain the second equality in (25). ☐

**Theorem** **6.** The target chain rule, identity property and local positivity, cannot be simultaneously satisfied.

**Proof.** Consider the probability distribution Tbc, and in particular, the isomorphic probability distributions $P(T_{1,2})$ and $P(T_{1,3})$. By the identity property,

(A35) $$\begin{array}{cccc} U(S_{1}\backslash S_{2}\to T_{1,2}) & =1\;\mathrm{bit}, & U(S_{2}\backslash S_{1}\to T_{1,2}) & =1\;\mathrm{bit}, \end{array}$$

and hence, $R(S_{1},S_{2}\to T_{1,2})$ = 0 bit. On the other hand, by local positivity,

(A36) $$\begin{array}{cccc} 1-1C(S_{1},S_{2}\to T_{3}) & =1\;\mathrm{bit}, & R(S_{1},S_{2}\to T_{1}|T_{3}) & =1\;\mathrm{bit} \end{array}$$

Then by the target chain rule,

(A37) $$\begin{array}{cccc} 1-1C(S_{1},S_{2}\to T_{1,3}) & =1\;\mathrm{bit} & R(S_{1},S_{2}\to T_{1,3}) & =1\;\mathrm{bit}, \end{array}$$

Finally, since $P(T_{1,2})$ is isomorphic to $P(T_{1,3})$ we have that, $R\left(S_{1},S_{2}\to T_{1,3}\right)=R\left(S_{1},S_{2}\to T_{1,2}\right)$, which is a contradiction. ☐

Theorem 6 can be informally generalised as follows: it is not possible to simultaneously satisfy the target chain rule, the identity property, and have only $C(S_{1},S_{2}\to T)$ = 1 bit in the probability distribution Xor without having negative (average) PI atoms in probability distributions where there is no ambiguity from any source. To see this, again consider decomposing the isomorphic probability distributions $P(T_{1,2})$ and $P(T_{1,3})$. In line with (A35), decomposing $T_{1,2}$ via the identity property yields $C(S_{1},S_{2}\to T_{1,2})$ = 0 bit. On the other hand, decomposing $T_{1,3}$ yields $C(S_{1},S_{2}\to T_{3})$ = 1 bit. Since $P(T_{1,2})$ is isomorphic to $P(T_{1,3})$, the target chain rule requires that,

(A38) $$\begin{array}{cccccc} C(S_{1},S_{2}\to T_{1}|T_{3}) & =-1\;\mathrm{bit}, & U(S_{1}\backslash S_{2}\to T_{1}|T_{3}) & =1\;\mathrm{bit}, & U(S_{2}\backslash S_{1}\to T_{1}|T_{3}) & =1\;\mathrm{bit}. \end{array}$$

That is, one would have to accept the negative (average) PI atom $C(S_{1},S_{2}\to T_{1}|T_{3})$ = −1 bit despite the fact that there are no non-zero pointwise ambiguity terms upon splitting any of $i(s_{1};t_{1}|t_{3})$, $i(s_{2};t_{1}|t_{3})$ and $i(s_{1,2};t_{1}|t_{3})$ into specificity and ambiguity. Although this does not constitute a formal proof that the identity property is incompatible with the target chain rule, one would have to accept and find a way to justify $C(S_{1},S_{2}\to T_{1}|T_{3})$ = −1 bit. Since there is no ambiguity in $i(s_{1};t_{1}|t_{3})$, $i(s_{2};t_{1}|t_{3})$ and $i(s_{1,2};t_{1}|t_{3})$, this result is not reconcilable within the framework of specificity and ambiguity.

## Appendix C. Additional Example Probability Distributions

### Appendix C.1. Probability Distribution *Tbep*

Figure A1 shows the probability distribution three bit–even parity (Tbep) which considers binary predictors variables $S_{1}$, $S_{2}$ and $S_{3}$ which are constrained such that together their parity is even. The target variable *T* is simply a copy of the predictors, i.e., $T=T_{1,2,3}=\left(T_{1},T_{2},T_{3}\right)$ where $T_{1}=S_{1}$, $T_{2}=S_{2}$ and $T_{3}=S_{3}$. (Equivalently, the target can be represented by any four state variable *T*.) It was introduced by Bertschinger et al. [5] and revisited by Rauh et al. [13] who (as mentioned in Section 5.5) used it to prove the following by counter-example: there is no measure of redundant average information for more than two predictor variables which simultaneously satisfies the Williams and Beer Axioms, the identity property, and local positivity. The measures $I_{\mathrm{red}}$, $\tilde{UI}$ and $S_{\mathrm{VK}}$ these properties. Hence, this probability distribution which has been used to demonstrate that these measures are not consistent with the PID framework in the general case of an arbitrary number of predictor variables.

This example is similar to Tbc in the several ways. Firstly, due to the symmetry in the probability distribution, each realisation will have the same pointwise decomposition. Secondly, there is an isomorphism between the probability distributions P(T) and $P(S_{1},S_{2},S_{3})$, and hence the pointwise ambiguity provided by any (individual or joint) predictor event is 0 bit (since given *t*, one knows $s_{1}$, $s_{2}$ and $s_{3}$). Thirdly, the individual predictor events $s_{1}$, $s_{2}$ and $s_{3}$ each exclude 12 of the total probability mass in P(T) and so each provide 1 bit of pointwise specificity. Thus, there is 1 bit of three-way redundant, pointwise specificity in each realisation. Fourthly, the joint predictor event $s_{1,2,3}$ excludes 34 of the total probability mass, providing 2 bit of pointwise specificity (which is similar to Tbc). However, unlike Tbc, one could consider the three joint predictor events $s_{1,2}$, $s_{1,3}$ and $s_{2,3}$. These joint pairs also exclude 34 of the total probability mass each, and hence also each provide 2 bit of pointwise specificity. As such, there is 1 bit of pointwise, three-way redundant, pairwise complementary specificity between these three joint pairs of source events, in addition to the 1 bit of three-way redundant, pointwise specificity. Finally, putting this together and averaging over all realisations, Tbep consists of 1 bit of three-way redundant information and 1 bit of three-way redundant, pairwise complementary information. The resultant average decomposition is the same as the decomposition induced by $I_{\min}$ [5].

### Appendix C.2. Probability Distribution *Unq*

Figure A2 shows the decomposition of the probability distribution *unique* (Unq). Note that this probability distribution corresponds to RdnErr where the error probability ε=12, and hence the similarity in the resultant distributions. The results may initially seem unusual, that the predictor $S_{1}$ is not uniquely informative since $U(S_{1}\backslash S_{2}\to T)$ = 0 bit as one might intuitively expect. Rather it is deemed to be redundantly informative RI = 1 bit with the predictor $S_{2}$ which is also uniquely misinformative $U(S_{2}\backslash S_{1}\to T)$ = −1 bit. This is because both $S_{1}$ and $S_{2}$ provide $I^{+}\left(S_{1}\to T\right)=I^{+}\left(S_{2}\to T\right)$ = 1 bit of specificity; however the information provided by $S_{2}$ is unique in that the 1 bit provided is not “useful” ([42], p. 21) and hence $I(S_{2}\to T)$ = 1 bit while $I(S_{2}\to T)$ = 1 bit. Finally, the complementary information $C(S_{1},S_{2}\to T)$ = 1 bit is required by the decomposition in order to balance this 1 bit of unique ambiguity. The results in this example partly explain our preference for term *complementary information* as opposed to *synergistic information*—while $C(S_{1},S_{2}\to T)$ = 1 bit is readily explainable, it would be dubious to refer to this as *synergy* given that $S_{1}$ enables perfect predictions of *T* without any knowledge of $S_{2}$.

### Appendix C.3. Probability Distribution *And*

Figure A3 shows the decomposition of the probability distribution *and* (And). Note that the probability distribution *or* (Or) has the same decomposition as the target distributions are isomorphic.

## References

[1] Williams P.L., Beer R.D. (2010). Information decomposition and synergy. Nonnegative decomposition of multivariate information. arXiv.

[B2-entropy-20-00297] 2.Williams, P.L.; Beer, R.D. Indiana University. DecomposingMultivariate Information. Privately communicated, 2010. This unpublished paper is highly similar to [1]. Crucially, however, this paper derives the redundancy lattice from the W&B Axioms 1–3 of Section 1. In contrast, [1] derives the redundancy lattice as a property of the particular measure *I*_min_.

[3] Olbrich E., Bertschinger N., Rauh J. (2015). Information decomposition and synergy. Entropy.

[4] Lizier J.T., Flecker B., Williams P.L. Towards a synergy-based approach to measuring information modification. Proceedings of the IEEE Symposium on Artificial Life (ALife).

[5] Bertschinger N., Rauh J., Olbrich E., Jost J. (2013). Shared information—New insights and problems in decomposing information in complex systems. Proceedings of the European Conference on Complex Systems.

[6] Harder M., Salge C., Polani D. (2013). Bivariate measure of redundant information. Phys. Rev. E.

[7] Griffith V., Chong E.K., James R.G., Ellison C.J., Crutchfield J.P. (2014). Intersection information based on common randomness. Entropy.

[8] Shannon C.E. (1948). A Mathematical Theory of Communication. Bell Syst. Tech. J..

[9] Fano R. (1961). Transmission of Information.

[10] Harder M. (2013). Information driven self-organization of agents and agent collectives. Ph.D. Thesis.

[11] Bertschinger N., Rauh J., Olbrich E., Jost J., Ay N. (2014). Quantifying unique information. Entropy.

[12] Griffith V., Koch C., Prokopenko M. (2014). Quantifying Synergistic Mutual Information. Guided Self-Organization: Inception.

[13] Rauh J., Bertschinger N., Olbrich E., Jost J. Reconsidering unique information: Towards a multivariate information decomposition. Proceedings of the 2014 IEEE International Symposium on Information Theory.

[14] Perrone P., Ay N. (2016). Hierarchical Quantification of Synergy in Channels. Front. Robot. AI.

[15] Griffith V., Ho T. (2015). Quantifying redundant information in predicting a target random variable. Entropy.

[16] Rosas F., Ntranos V., Ellison C.J., Pollin S., Verhelst M. (2016). Understanding interdependency through complex information sharing. Entropy.

[17] Barrett A.B. (2015). Exploration of synergistic and redundant information sharing in static and dynamical Gaussian systems. Phys. Rev. E.

[18] Ince R. (2017). Measuring Multivariate Redundant Information with Pointwise Common Change in Surprisal. Entropy.

[19] Ince R.A. (2017). The Partial Entropy Decomposition: Decomposing multivariate entropy and mutual information via pointwise common surprisal. arXiv.

[20] Chicharro D., Panzeri S. (2017). Synergy and Redundancy in Dual Decompositions of Mutual Information Gain and Information Loss. Entropy.

[21] Rauh J., Banerjee P.K., Olbrich E., Jost J., Bertschinger N. (2017). On Extractable Shared Information. Entropy.

[22] Rauh J., Banerjee P.K., Olbrich E., Jost J., Bertschinger N., Wolpert D. (2017). Coarse-Graining and the Blackwell Order. Entropy.

[23] Rauh J. (2017). Secret sharing and shared information. Entropy.

[24] Faes L., Marinazzo D., Stramaglia S. (2017). Multiscale information decomposition: Exact computation for multivariate Gaussian processes. Entropy.

[25] Pica G., Piasini E., Chicharro D., Panzeri S. (2017). Invariant components of synergy, redundancy, and unique information among three variables. Entropy.

[26] James R.G., Crutchfield J.P. (2017). Multivariate dependence beyond shannon information. Entropy.

[27] Makkeh A., Theis D.O., Vicente R. (2017). Bivariate Partial Information Decomposition: The Optimization Perspective. Entropy.

[28] Kay J.W., Ince R.A., Dering B., Phillips W.A. (2017). Partial and Entropic Information Decompositions of a Neuronal Modulatory Interaction. Entropy.

[29] Angelini L., de Tommaso M., Marinazzo D., Nitti L., Pellicoro M., Stramaglia S. (2010). Redundant variables and Granger causality. Phys. Rev. E.

[30] Stramaglia S., Angelini L., Wu G., Cortes J.M., Faes L., Marinazzo D. (2016). Synergetic and redundant information flow detected by unnormalized Granger causality: Application to resting state fMRI. IEEE Trans. Biomed. Eng..

[31] Ghazi-Zahedi K., Langer C., Ay N. (2017). Morphological computation: Synergy of body and brain. Entropy.

[32] Maity A.K., Chaudhury P., Banik S.K. (2017). Information theoretical study of cross-talk mediated signal transduction in MAPK pathways. Entropy.

[33] Tax T., Mediano P.A., Shanahan M. (2017). The partial information decomposition of generative neural network models. Entropy.

[34] Wibral M., Finn C., Wollstadt P., Lizier J.T., Priesemann V. (2017). Quantifying Information Modification in Developing Neural Networks via Partial Information Decomposition. Entropy.

[35] Woodward P.M. (1953). Probability and Information Theory: With Applications to Radar.

[36] Woodward P.M., Davies I.L. (1952). Information theory and inverse probability in telecommunication. Proc. IEE-Part III Radio Commun. Eng..

[37] Gray R.M. (1988). Probability, Random Processes, and Ergodic Properties.

[38] Martin N.F., England J.W. (1984). Mathematical Theory of Entropy.

[39] Finn C., Lizier J.T. (2018). Probability Mass Exclusions and the Directed Components of Pointwise Mutual Information. arXiv.

[40] Kelly J.L. (1956). A new interpretation of information rate. Bell Labs Tech. J..

[41] Ash R. (1965). Information Theory.

[42] Shannon C.E., Weaver W. (1998). The Mathematical Theory of Communication.

[43] Cover T.M., Thomas J.A. (2012). Elements of Information Theory.

[44] Pearl J. (1988). Probabilistic Reasoning in Intelligent Systems: Networks of Plausible Inference.

[45] Rota G.C. (1964). On the foundations of combinatorial theory I. Theory of Möbius functions. Probab. Theory Relat. Field.

[46] Stanley R.P. (2012). Enumerative Combinatorics. Cambridge Studies in Advanced Mathematics.

[47] Davey B.A., Priestley H.A. (2002). Introduction to Lattices and Order.

[48] Ross S.M. (2009). A First Course in Probability.

